# Immune checkpoint inhibitor integration in locoregionally advanced nasopharyngeal carcinoma: a prospective evidence synthesis on efficacy, safety, and therapeutic optimization

**DOI:** 10.3389/fimmu.2026.1840292

**Published:** 2026-06-26

**Authors:** Peng Chen, Ying Jiang, Chen Han, Lian Jian, Yu Gong, Hui Xie, Ziying Zhang, Yaqian Han

**Affiliations:** 1 Department of Diagnostic Radiology, The Affiliated Cancer Hospital of Xiangya School of Medicine, Central South University/Hunan Cancer Hospital, Changsha, Hunan, China; 2 Department of Radiation Oncology, The Affiliated Cancer Hospital of Xiangya School of Medicine, Central South University/Hunan Cancer Hospital, Changsha, Hunan, China; 3 School of Basic Medicine, Hubei University of Chinese Medicine, Wuhan, Hubei, China; 4 Department of Radiology, State Key Laboratory of Oncology in South China, Guangdong Provincial Clinical Research Center for Cancer, Sun Yat-sen University Cancer Center, Guangzhou, China

**Keywords:** chemoimmunotherapy, immune checkpoint inhibitors, locoregionally advanced nasopharyngeal carcinoma, meta-analysis, treatment sequencing

## Abstract

**Background:**

Several prospective and randomized trials support immune checkpoint inhibitors (ICIs) as an emerging component of standard definitive treatment for locoregionally advanced nasopharyngeal carcinoma (LA-NPC). The key uncertainty has shifted from whether ICIs have antitumor activity to how they should be incorporated into multimodality therapy, including therapeutic modality, treatment timing, chemotherapy backbone, treatment duration, and agent selection.

**Methods:**

We performed a pooled analysis of seven prospective trials enrolling 1,788 patients with LA-NPC, with data retrieved through December 2025. ICI-based regimens were evaluated across four prespecified dimensions: therapeutic modality, immunotherapy timing, chemotherapy backbone, and individual ICI agent. The protocol was registered with PROSPERO (CRD420261293069).

**Results:**

Seven prospective trials involving 1788 patients were included. ICI-containing strategies were associated with high pooled survival estimates, including 3-year overall survival (OS) of 96.4% and 3-year failure-free survival (FFS) of 88.2%. Exploratory strategy-level analyses suggested clinically relevant differences across ICI integration approaches. In the neoadjuvant setting, chemoimmunotherapy plus antiangiogenic therapy showed the highest complete response (CR) estimate (26.5%) but also the highest grade 3 or higher treatment-related adverse event estimate (65.3%); ICI monotherapy showed limited neoadjuvant activity, with a CR estimate of 1.0%. For treatment sequencing, neoadjuvant-adjuvant ICI administration showed favorable long-term disease-control estimates, including 3-year locoregional recurrence-free survival of 96.0% and 3-year OS of 99.0%, whereas full-course neoadjuvant-concurrent-adjuvant ICI administration was associated with the lowest progressive disease estimate (0.2%). Chemotherapy backbone also appeared relevant: modified taxane-platinum-fluoropyrimidine showed a higher neoadjuvant CR estimate than gemcitabine-cisplatin (31.4% vs 13.7%). At the agent level, camrelizumab-based regimens achieved neoadjuvant objective response of 100.0%, although with a higher immune-related adverse event estimate (81.8%); toripalimab-based regimens showed 3-year OS of 97.0% with a lower overall immune-related adverse event estimate (53.6%).

**Conclusion:**

ICI-containing strategies showed favorable current survival estimates and measurable tumor activity in LA-NPC; however, their clinical relevance appears to vary according to how ICIs are integrated into multimodality treatment. Given that several strategy-level estimates were based on few heterogeneous studies and relatively short follow-up, these findings should be viewed as hypothesis-generating and used to inform treatment optimization and future randomized, biomarker-informed trials, rather than to identify a single preferred ICI strategy.

**Systematic review registration:**

https://www.crd.york.ac.uk/PROSPERO/view/CRD420261293069, identifier CRD420261293069.

## 1 Introduction

Nasopharyngeal carcinoma (NPC), a malignancy arising from the nasopharyngeal mucosal epithelium, exhibits marked geographic and ethnic variation in incidence, with the highest burden concentrated in Southern China and other parts of Southeast Asia (1, 2). Globally, an estimated 130,000 new cases are diagnosed annually, of which over 70% present with locoregionally advanced disease (LA-NPC; AJCC 8th edition stage III-IVA) (1). For non-metastatic LA-NPC, contemporary guideline-based management remains centered on intensity-modulated radiotherapy combined with platinum-based chemotherapy, with the sequencing of systemic therapy tailored according to stage, disease burden, and recurrence risk. The ESMO-EURACAN Clinical Practice Guideline for NPC covers diagnosis, treatment, and follow-up, and its subsequent update highlights adjuvant capecitabine after chemoradiotherapy as an option that improves progression-free survival (PFS) for high-risk LA-NPC. The NCCN Head and Neck Cancers Guidelines Insights summarizes the most recent NCCN recommendations for nasopharynx cancer, including stage-adapted radiotherapy, indications for systemic therapy, and ongoing research in treatment optimization. The CSCO-ASCO guideline specifically addresses definitive-intent treatment for stage II-IVA NPC and provides evidence-based recommendations on radiotherapy technique and the use of concurrent, induction, and adjuvant chemotherapy in combination with radiotherapy (7–10). In contemporary practice, induction chemotherapy (IC) followed by concurrent chemoradiotherapy (CCRT) remains widely used for LA-NPC and has yielded commendable 5-year locoregional control rates exceeding 90% and overall survival (OS) rates above 80% (3, 4). Despite these gains, approximately 20-30% of patients experience disease recurrence or distant metastasis, which carries a substantially worse prognosis (4). Moreover, long-term survivors frequently bear the burden of severe late treatment-related toxicities, including radiation-induced fibrosis, sensorineural hearing loss, and temporal lobe necrosis, that meaningfully impair quality of life (4, 5). Improving disease control while limiting treatment-related morbidity therefore remains a central challenge in the contemporary management of LA-NPC (6).

Cisplatin-based combination regimens, most notably gemcitabine plus cisplatin (GP) and docetaxel-cisplatin-fluorouracil (TPF), constitute the backbone of IC, with the dual aims of achieving locoregional tumor debulking and eradicating subclinical micrometastatic disease prior to definitive CCRT (3, 10–12). Nevertheless, a clinically meaningful proportion of patients, approximately 20-30%, exhibit suboptimal responses to IC (stable or progressive disease), a finding consistently associated with inferior long-term outcomes (13, 14). The critical importance of maximizing early tumor regression has been recently underscored by a landmark multicenter randomized phase III trial demonstrating that radiotherapy targeted to the reduced post-IC tumor volume achieved equivalent locoregional control to conventional full-volume irradiation, while significantly reducing severe late toxicity (15). This finding establishes a compelling rationale: maximizing depth of tumor regression during IC directly enables treatment de-escalation strategies that can improve both survival outcomes and quality of life. However, the optimal approach to enhancing tumor regression, particularly through the integration of immunotherapy, remains incompletely defined, partly owing to substantial heterogeneity in regimen composition across contemporary trials.

NPC has several immunologic features that make ICI integration biologically plausible, including Epstein-Barr virus association, lymphocyte-rich tumor stroma, an immunologically active but suppressive tumor microenvironment, and frequent PD-L1 expression on tumor or immune cells (1, 4). Recent reviews and multi-omics studies have further emphasized that the Epstein-Barr virus-associated tumor microenvironment, drug-resistance pathways, and tumor-intrinsic molecular regulators may shape NPC biology and therapeutic sensitivity (16, 17). These features provide a rationale for combining checkpoint blockade with chemotherapy and radiotherapy in definitive treatment (4). More broadly, contemporary reviews across cancer types have summarized the biological roles of immune checkpoint molecules, mechanisms of resistance to immune checkpoint blockade, and the expanding clinical application of ICIs in selected solid tumors (18–20). Clinical evidence has also moved beyond early activity signals. In the neoadjuvant setting, camrelizumab combined with apatinib and induction chemotherapy showed encouraging outcomes in patients with stage N3 disease (21).

In the adjuvant setting, the DIPPER trial showed improved event-free survival (EFS) with camrelizumab after induction chemotherapy and concurrent chemoradiotherapy (22). In a full-course strategy, CONTINUUM showed improved EFS when sintilimab was added during induction chemotherapy, concurrent chemoradiotherapy, and adjuvant treatment (23). DIAMOND evaluated a different approach, showing that a toripalimab-based strategy without concurrent cisplatin may preserve disease control while reducing treatment burden in selected patients (24).

The accumulating prospective data also highlight an important unresolved issue: ICI-containing regimens in LA-NPC are not a single therapeutic approach. Available studies differ in treatment modality, timing of ICI administration, duration of exposure, chemotherapy backbone, use of antiangiogenic or other targeted agents, and the specific ICI used (21–24, 29–31). These differences complicate cross-trial interpretation and may affect the balance among early tumor regression, durable disease control, treatment-related toxicity, and quality of life. Therefore, we performed a systematic review and meta-analysis of prospective studies to summarize contemporary tumor activity, survival, and safety estimates of ICI-containing strategies in LA-NPC and to examine how regimen composition, ICI timing, chemotherapy backbone, and agent selection may be associated with clinical outcomes.

## 2 Methods

This systematic review and meta-analysis were conducted in accordance with the Preferred Reporting Items for Systematic Reviews and Meta-Analyses (PRISMA) guidelines, based on a predefined protocol (25, 26).

### 2.1 Search strategy

A comprehensive and systematic literature search was conducted across five databases, namely PubMed, EMBASE, Web of Science, the Cochrane Library, and the China National Knowledge Infrastructure (CNKI), covering all records up to December 30, 2025, without language restrictions. Reference lists of all retrieved articles were additionally screened by hand to identify potentially eligible studies not captured by the electronic search. The detailed search strategy, including all Boolean operators and MeSH terms employed, was provided in **Supplementary Table 1**.

### 2.2 Study selection and eligibility criteria

Eligibility criteria were defined *a priori* using the PICO (Population, Intervention, Comparison, Outcome) framework. Studies were included if they enrolled adult patients (aged ≥18 years) with newly diagnosed LA-NPC and evaluated treatment regimens containing one or more ICIs, administered either as monotherapy or in combination with chemotherapy, targeted therapy, or radiotherapy. Eligible studies were required to report at least one predefined tumor activity, survival, disease-control, or safety outcome, including objective response rate (ORR), RECIST response categories of complete response (CR), partial response (PR), stable disease (SD), and progressive disease (PD), OS, PFS, EFS, failure-free survival (FFS), distant metastasis-free survival (DMFS), locoregional recurrence-free survival (LRRFS), adverse events (AEs), treatment-related adverse events (TRAEs), or immune-related adverse events (irAEs). Studies were excluded if they focused exclusively on recurrent or metastatic NPC, reported duplicate data from previously published cohorts, or employed non-prospective study designs, including retrospective analyses, systematic reviews, case reports, letters to the editor, and commentaries.

### 2.3 Data extraction

Two investigators independently extracted data using a pre-standardized extraction form encompassing study characteristics, patient demographics and baseline clinical features, full details of the intervention (ICI agent, timing of immunotherapy, and combination strategy), and all pre-specified clinical endpoints. Discrepancies between reviewers were resolved through structured discussion and consensus; in cases of persistent disagreement, arbitration by a senior reviewer was sought.

### 2.4 Quality assessment

The methodological quality of randomized controlled trials (RCTs) was evaluated using the Cochrane Risk of Bias Tool, which assesses seven predefined bias domains including sequence generation, allocation concealment, blinding of participants and outcome assessors, completeness of outcome data, and selective outcome reporting (27). For non-randomized and single-arm prospective studies, the ROBINS-I tool was applied to evaluate potential bias across domains including confounding, participant selection, classification of interventions, and outcome measurement (28). All quality assessments were performed independently by two reviewers blinded to study authorship and journal of publication; discrepancies were resolved by consensus or third-party consultation.

### 2.5 Statistical analysis

Endpoints were organized according to their role in clinical interpretation rather than treated as separate confirmatory hypotheses. The principal clinical outcome for interpretation was 3-year OS. Key secondary disease-control outcomes included 3-year FFS, EFS, DMFS, LRRFS, and 2-year PFS, as defined in the original trials. These survival endpoints were reported separately because they reflect overlapping but nonidentical aspects of disease control and were not interpreted as independent tests of treatment benefit.

Tumor activity outcomes, including ORR and RECIST response categories (CR, PR, SD, and PD), were summarized descriptively. Safety outcomes were described as overall regimen-level toxicity, including AEs or TRAEs by severity grade, and immune-specific toxicity, including irAEs. This distinction was retained because the included ICI-containing strategies differed in chemotherapy backbone, radiotherapy context, targeted therapy use, treatment duration, and ICI agent. Overall AEs or TRAEs and irAEs were therefore interpreted as complementary measures of tolerability rather than independent confirmatory endpoints.

Pooled estimates with corresponding 95% confidence intervals (CIs) were calculated using random-effects models when substantial heterogeneity was present. Statistical heterogeneity was assessed using Cochran’s Q test and the I² statistic. Subgroup analyses according to treatment modality, ICI timing, chemotherapy backbone, treatment duration, and individual ICI agent were considered exploratory and hypothesis-generating. Given the descriptive purpose of this evidence synthesis, the limited number of eligible prospective studies, and the clinical correlation among several outcomes, no formal multiplicity adjustment was applied. Accordingly, nominal differences across response categories, survival measures, toxicity outcomes, and subgroups were interpreted cautiously and were not used to establish statistical superiority among ICI integration strategies. Sensitivity analyses were conducted by sequentially omitting individual studies to assess the robustness of pooled estimates. Publication bias was evaluated through visual inspection of funnel plots and Egger’s regression test when sufficient studies were available. All analyses were performed using Stata version 15.0, and two-sided P values below 0.05 were considered nominally significant.

## 3 Results

### 3.1 Systematic review and study characteristics

**Figure 1:**
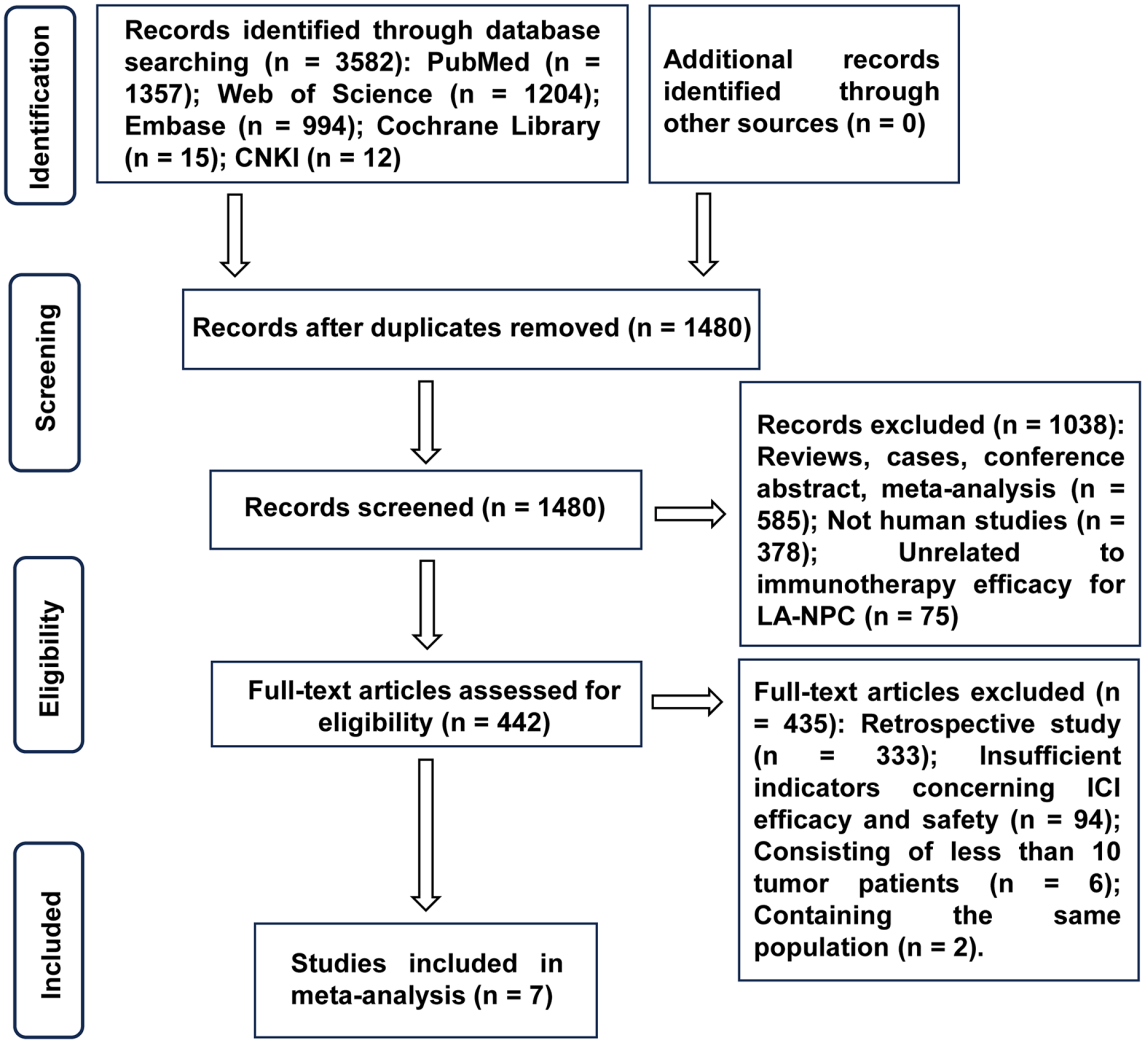
Flow diagram of the literature search and study selection.

Our systematic literature search, conducted in accordance with PRISMA guidelines, identified 3,582 records in total, from which 442 full-text articles were retrieved for detailed eligibility assessment (**Figure 1**). Following application of the predefined inclusion and exclusion criteria, seven prospective clinical trials enrolling 1,788 patients with LA-NPC and published up to December 30, 2025, were included in the final meta-analysis (21–24, 29–31). As detailed in **Table 1**, all included studies focused on stage III-IV disease and enrolled patients from Chinese institutions. The study pool comprised four RCTs (22–24, 29) and three non-randomized prospective studies (21, 30, 31); among these, four employed a two-arm comparative design (22–24, 29). By phase designation, four trials were phase II (21, 29–31) and three were phase III (22–24).

**Table 1:** Baseline characteristics and treatment regimens of the included prospective trials.

First author, year	Country/ Region	Trial name	Inclusion criteria	Trial phase	Trial design	Total number, n	Male, n(%)	Median age, y (range)	T stage, n(%)	N stage, n(%)	PD-L1 CPS < 20, n(%)	PD-L1 CPS >=20, n(%)	PD-L1 CPS unknown, n(%)	Pretreatment EBV DNA, n(%)		Median pretreatment EBV DNA (copies/mL)	EBV DNA before CRT, n(%)	EBV DNA after CRT, n(%)
Liu 2024 (29)	China	NCT03925090	High-risk stage III-IVa LA-NPC AJCC8th	II	Randomised, single-centre, double-blind, placebo-controlled	100	78(78)	47(19-65)	T2:8(8);T3:71(71);T4:21(21)	N0:2(2);N1:29(29);N2:29(29);N3:40(40)	13(13)	72(72)	15(15)	EBV DNA < 4000: 54(54)	EBV DNA > 4000: 46(46)	3815	Detectable 83(83); Undetectable 17(17)	Detectable 5(5); Undetectable 95(95)
						50	37(74)	46.5(20-65)	T2:3(6);T3:38(76);T4:9(18)	N1:16(32);N2:12(24);N3:22(44)	7(14)	34(68)	9(18)	EBV DNA < 4000: 25(50)	EBV DNA > 4000: 25(50)	3980	Detectable 46(92); Undetectable 3(6)	Detectable 8(16); Undetectable 41(82)
Liang 2024 (21)	China	CHICTR2000032317	TanyN3M0 NPC	II	Single-center, single-arm	49	37(75.5)	46(37-52)	T1:3(6.1);T2:7(14.3);T3:26(53.1);T4:13(26.5)	All N3	\	\	\	Detectable 48/49 (98.0)	\	4710	Undetectable 9/48 (19)	Detectable 2(4); Undetectable 46(96)
Liu 2024 (23)	China	NCT03700476	High-risk non-metastatic stage III-IVa LA-NPC (excluding T3-4N0 and T3N1) AJCC8th	III	Randomised, multicentre, open-label, parallel-group, controlled	210	158(75)	46(38-53)	T1:6(3);T2:21(10);T3:87(41);T4:96(46)	N1:43(20);N2:95(45);N3:72(34)	CPS<1: 20(10) CPS>=1: 110(52) CPS unknown: 80(38)		\	\		\	\	\
						215	155(72)	46(40-53)	T1:5(2);T2:26(12);T3:78(36);T4:106(49)	N1:48(22);N2:100(47);N3:67(31)	CPS<1:22(10) CPS>=1:105(49) CPS unknown:88(41)		\	\		\	\	\
Yu 2025 (30)	China	ChiCTR240008603	T1-4N2-3M0 LA-NPC AJCC8th	II	Investigator-initiated, single-arm, open-label	30	26(86.7)	49.5	T1:2(6.7);T2:5(16.7);T3:17(56.7);T4:6(20)	N2:19(63.3);N3:11(36.7)	2(6.7)	28(93.3)	\	EBV DNA < 4000: 18(60)	EBV DNA > =4000:12(40)	3130	Detectable 8/29(27.6)	\
Liang 2025 (22)	China	NCT03427827	T4N1M0 or T1-4N2-3M0 LA-NPC AJCC8th	III	Randomized,open-label, multicenter	226	172(76.1)	46(18-65)	T1:12(5.3);T2:15(6.7);T3:97(42.9);T4:102(45.1)	N1:40(17.7);N2:107(47.3);N3:79(35)	98(43.4)	34(15)	94(41.6)	EBV DNA+:16(7.1); EBV DNA-:190(84.1); EBV DNA not tested:20(8.8)		\	\	\
						224	168(75)	46(19-65)	T1:7(3.1);T2:27(12.1);T3:85(37.9);T4:105(46.9)	N1:48(21.4);N2:113(50.5);N3:63(28.1)	99(44.2)	34(15.2)	91(40.6)	EBV DNA+:25(11.1); EBV DNA-:189(84.4); EBV DNA not tested:10(4.5)		\	\	\
Xu 2025 (31)	China	NCT03984357	Stages III-IVa NPC AJCC8th	II	Multicenter, single-arm	152	124(81.6)	49(39-56)	T1:4(2.6);T2:20(13.2);T3:61(40.1);T4:67(44.1)	N1:26(17.1);N2:74(48.7);N3:52(34.2)	60(57.1)	37(38.1)	55(36.2)	EBV DNA<1000: 58(47.9); EBV DNA >1000: 63(52.1)		\	Undetectable 82.6%	Undetectable 95.0%
Xu 2025 (24)	China	NCT04907370	Stages T4N1M0 or T1-4N2-3M0 AJCC8th	III	Randomized, open-label, multicenter	266	198(74.4)	47(39-54)	T1:7(2.6);T2:33(12.4);T3:111(41.7);T4:115(43.2)	N1:47(17.7);N2:122(45.9); N3:97(36.5)	CPS<1: 31(11.7) CPS 1-19: 57(21.4)	34(12.8)	144(54.1)	EBV DNA+: 215(80.8); EBV DNA-: 43(16.2); EBV DNA not available: 8(3.0)		\	Undetectable 165/226 (73.0)	Undetectable 144/164 (87.8)
						266	200(75.2)	47(38-54)	T1:7(2.6);T2:32(12.0);T3:112(42.1); T4:115(43.2)	N1:49(18.4);N2:120(45.1);N3:97(36.5)	CPS<1: 38(14.3) CPS 1-19: 56(21.1)	40(15.0)	132(49.6)	EBV DNA+: 224(84.2); EBV DNA-: 36(13.5); EBV DNA not available: 6(2.3)		\	Undetectable 165/230 (71.7)	Undetectable 133/157 (84.7)

The included therapeutic regimens were classified into four principal categories based on treatment modality and timing. Two studies investigated neoadjuvant chemoimmunotherapy: one utilizing a nab-paclitaxel, cisplatin, and capecitabine (TPC) regimen (21) and the other employing nab-paclitaxel, cisplatin, and S-1 (30), with both protocols administering three neoadjuvant cycles prior to CCRT. Adjuvant immunotherapy was evaluated in one trial, in which camrelizumab (12 cycles) was compared with capecitabine following standard induction and concurrent chemoradiotherapy (22). A combined neoadjuvant and adjuvant immunotherapy strategy was assessed in one study, utilizing a placebo-controlled design with two cycles of neoadjuvant and eight cycles of adjuvant toripalimab (29). The remaining three trials evaluated comprehensive, full-course regimens encompassing neoadjuvant, concurrent, and adjuvant ICI administration: one compared a chemoimmunotherapy combination to GP alone (23); a single-arm trial assessed nivolumab integrated throughout GP induction, radiotherapy, and the adjuvant phase (31) and one trial compared concurrent radiotherapy with or without cisplatin in the context of toripalimab-based immunotherapy (24). Within the neoadjuvant component specifically, five studies investigated chemoimmunotherapy combinations employing TPC (21), TPF (30), and GP (23, 24, 31) backbones, while one study evaluated immunotherapy monotherapy as the neoadjuvant component (29). The ICI agents used across the included trials were camrelizumab, toripalimab, sintilimab, and nivolumab. A comprehensive summary of efficacy and safety outcomes was provided in **Tables 2**, **3**.

**Table 2:** Tumor activity and survival endpoint data extracted from the included trials.

First author, year	Total number	Intervention arm	Control arm	Immunotherapy type	Immunotherapy timing	Immunotherapy cycles	Following therapy	Response to neoadjuvant therapy, n(%)	ORR after neoadjuvant therapy (%)	Response to treatments	Median follow-up (months)	PFS rate (%)	FFS rate (%)	EFS rate (%)	OS rate (%)	LRRFS rate (%)	DMFS rate (%)
Liu 2024 (29)	100	Toripalimab	\	Toripalimab	Neoadjuvant and adjuvant	2 + 8	Concurrent chemoradiotherapy+toripalimab maintenance	CR1(1); PR16(16); SD82(82); PD1(1)	17.0	CR97(97);PR1(1);SD0;PD2(2);CNBA0	37.9	2-y: 92.0 (86.7-97.3)	\	\	3-y: 99(97·0-100.0)	3-y: 96.3(88.5-98.8)	3-y: 90.4(81.5-95.0)
	50	\	Placebo	\	\	\	Concurrent chemoradiotherapy	CR0; PR1(2); SD48(96); CNBA:1(2)	2	CR45(90);PR0;SD0;PD4(8)CNBA1(2)	37.2	2-y: 74.0 (61.8-86.2)	\	\	3-y: 90·0(81·8-98·2)	3-y: 83.0(64.3-91.9)	3-y: 79.8(61.1-89.5)
Liang 2024 (21)	49	Camrelizumab+apatinib+TP+capecitabine	\	Camrelizumab	Neoadjuvant	3	Concurrent chemoradiotherapy	CR13(26.5); PR36(73.5)	49/49(100)	CR45(95.7);PR1(2.1);PD1(2.1)	28.7	2-y: 95.9	2-y: 95.9(84.9-98.9)	\	2-y: 98.0(87.6-99.7)	2-y: 97.9(86.9-99.7)	1-y: 98.0 (87.6-99.7) 2-y: 98.0 (87.6-99.7)
Liu 2024 (23)	210	Sintilimab with GP+RT with concurrent sintilimab+ adjuvant sintilimab	\	Sintilimab	Neoadjuvant, concurrent and adjuvant	3 + 3+6	\	CR23/209(11);PR169/209(81);SD11/209(5);PD0;CNBA:6(3)	\	CR193/209(92);PR5/209(2);SD1/209(1);PD4/209(2);CNBA:6(3)	41.9	\	\	3-y: 86(81-90)	3-y: 92(87-95)	3-y: 93(89-96)	3-y: 90(85-94)
	215	\	GP	\	\	\	CRT	CR24/214(11);PR170/214(79);SD17/214(8);PD0;CNBA:3(1)	\	CR198/214(93);PR12/214(6);SD1/214(1);PD1/214(1);CNBA:2(1)	41.9	\	\	3-y: 76.0(70-81)	3-y: 92(88-95)	3-y: 86(81-91)	3-y: 83(77-87)
Yu 2025 (30)	30	Camrelizumab+ modified TPF (nab-paclitaxel, cisplatin and S-1)	\	Camrelizumab	Neoadjuvant	3	CRT	CR12(41.4);PR17(58.6);SD0;PD0	100	\	\	\	\	\	\	\	\
Liang 2025 (22)	226	Camrelizumab	\	Camrelizumab	Adjuvant	12	\	\	\	\	39	\	\	3-y: 86.9 (82.6-91.5)	3-y: 96.4 (93.9-98.9)	3-y: 92.8 (89.3-96.4)	3-y: 92.4 (88.9-95.9)
	224	\	Capecitabine	\		\	\	\	\	39	\	\	3-y: 77.3 (71.9-83.1)	3-y: 92.9 (89.5-96.5)	3-y: 87.0 (82.5-91.7)	3-y: 84.5 (79.8-89.6)
Xu 2025 (31)	152	Nivolumab with GP+RT with concurrent nivolumab+adjuvant nivolumab	\	Nivolumab	Neoadjuvant, concurrent and adjuvant	3 + 3+6	\	CR23(15.1);PR111(73);SD12(7.9);PD1(0.7);CNBA5(3.3)	\	CR133(87.5);PR15(9.9);CNBA4(2.6)	43	3-y: 88.5	3-y: 88.5 (83.4-93.8)	\	3-y: 97.9 (95.6–100.0)	3-y: 93.0 (88.9-97.3)	3-y: 93.8 (90.0-97.8)
Xu 2025 (24)	266	Toripalimab with GP+RT with concurrent toripalimab+ adjuvant toripalimab	\	Toripalimab	Neoadjuvant, concurrent and adjuvant	3 + 3+11	\	CR39(14.7);PR196(73.7);SD24(9.0);PD0(0.0)	\	CR234(88.0);PR23(8.6);SD3(1.1);PD0(0.0)	37	\	3-y: 88.3(84.4-92.2)	\	3-y: 96.1(93.7-98.5)	3-y: 92.9(89.8-96.0)	3-y: 93.2(90.1-96.3)
	266	\	Toripalimab with GP+RT with concurrent cisplatin and toripalimab+adjuvant toripalimab		3 + 3+11	\	CR38(14.3);PR199(74.8);SD25(9.4);PD0(0.0)	\	CR237(89.1);PR17(6.4);SD2(0.8);PD0(0.0)	\		3-y: 87.6(83.5-91.7)	\	3-y: 96.5(94.1-98.9)	3-y: 93.6(90.7-96.5)	3-y: 91.6(88.1-95.1)

**Table 3:** AE outcome data of included trials.

First author, year	Total number	Intervention arm	Control arm	Immunotherapy type	Immunotherapy timing	Immunotherapy cycles	AEs, grade 1-2 n(%)	AEs >= grade 3, n(%)	irAEs, n(%)	irAEs, grade 1-2 n(%)	irAEs >= grade 3, n(%)
Liu 2024 (29)	100	Toripalimab	\	Toripalimab	Neoadjuvant and adjuvant	2+8	26(26)	74(74)	52(52)	42(42)	10(10)
	50	\	Placebo	\	\	\	16(32)	34(68)	\	11(22)	0
Liang 2024 (21)	49	Camrelizumab+apatinib+TP+capecitabine	\	Camrelizumab	Neoadjuvant	3	17(34.7)	32(65.3)	\	\	\
Liu 2024 (23)	210	Sintilimab with GP+RT with concurrent sintilimab+ adjuvant sintilimab	\	Sintilimab	Neoadjuvant, concurrent and adjuvant	3+3+6	52(25)	157(75)	125(60)	103(49)	22(11)
	215	\	GP	\	\	\	73(35)	140(65)	\	\	\
Yu 2025 (30)	30	Camrelizumab+ modified TPF (nab-paclitaxel, cisplatin and S-1)	\	Camrelizumab	Neoadjuvant	3	22/30(73.3)	8/30(26.6)	20/30(66.7)	19/30	1/30
Liang 2025 (22)	226	Camrelizumab	\	Camrelizumab	Adjuvant	12	176(85.9)	23(11.2)	188(91.7)	180	8(3.9)
	224	\	Capecitabine	\	\	\	182(82.3)	7(3.2)	\	\	\
Xu 2025 (31)	152	Nivolumab with GP+RT with concurrent nivolumab+adjuvant nivolumab	\	Nivolumab	Neoadjuvant, concurrent and adjuvant	3+3+6	91(59.8)	61(40.2)	\	\	\
Xu 2025 (24)	266	Toripalimab with GP+RT with concurrent toripalimab+ adjuvant toripalimab	\	Toripalimab	Neoadjuvant, concurrent and adjuvant	3+3+11	124(47.7)	136(52.3)	139(53.5)	126(48.5)	13(5.0)
	266	\	Toripalimab with GP+RT with concurrent cisplatin and toripalimab+ adjuvant toripalimab	\	\	3+3+11	95(36.4)	166(63.6)	148(56.7)	126(48.3)	22(8.4)

### 3.2 Quality assessment

Among the four included RCTs, all trials met core methodological standards, including appropriate randomization procedures, complete reporting of outcome data, and absence of clear selective outcome reporting. A shared limitation across these trials was that blinding of outcome assessors was not explicitly described in several instances, representing a potential source of detection bias. A comprehensive summary of RCT quality assessment is provided in **Supplementary Figure 1A**. For the three non-randomized and single-arm prospective studies, application of the ROBINS-I tool revealed an overall moderate level of methodological quality; most studies demonstrated low risk of bias in key domains, including control for confounding and fidelity to planned interventions. Full details were provided in **Supplementary Figure 1B**.

### 3.3 Pooled tumor activity analysis

**Figure 2:**
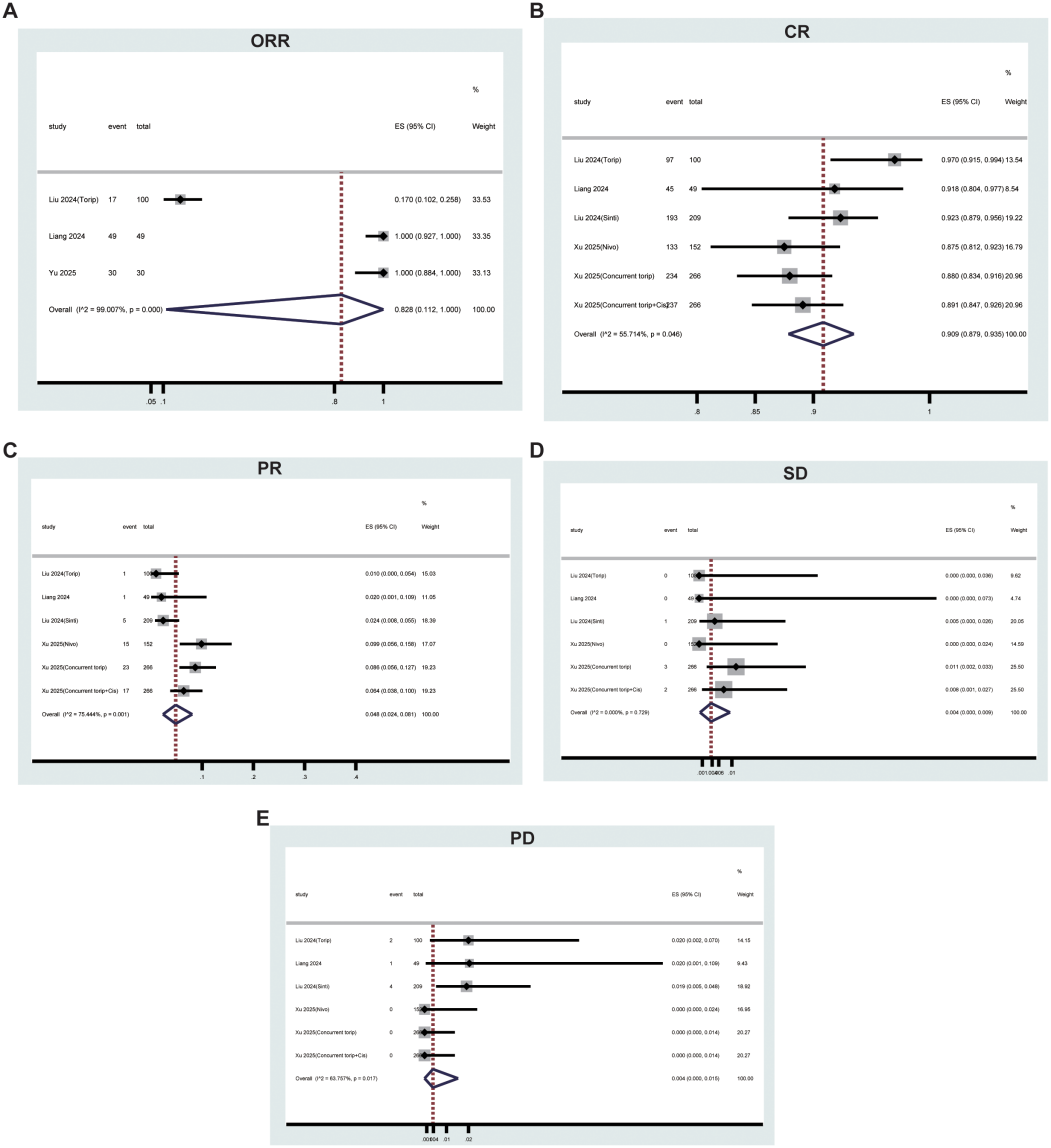
Forest plots of tumor activity outcomes in patients with LA-NPC treated with ICI-containing strategies. **(A)** ORR after neoadjuvant ICI-containing therapy; **(B)** CR, **(C)** PR, **(D)** SD, and **(E)** PD after completion of the full treatment regimen. LA-NPC, locoregionally advanced nasopharyngeal carcinoma; ICI, Immune checkpoint inhibitor; CR, complete response; PR, partial response; SD, stable disease; PD, progressive disease; ORR, objective response rate.

Among patients who received neoadjuvant therapy, CR rates across six studies ranged from 1.0% to 40.0%, yielding a pooled estimate of 14.4% (95% CI: 8.7-21.3%; I² = 86.9%). PR rates ranged from 16.0% to 80.9%, with a pooled value of 64.6% (95% CI: 48.9-78.9%; I² = 96.1%). SD was observed in 0-82.0% of patients, corresponding to a pooled rate of 11.4% (95% CI: 1.4-28.4%; I² = 97.9%), while PD ranged from 0% to 9.4%, yielding a pooled estimate of 0.7% (95% CI: 0.0-3.7%; I² = 89.2%) (**Supplementary Figures 2A–D**). The ORR, available from three studies, ranged from 17.0% to 100%, producing a pooled estimate of 82.8% (95% CI: 11.2-100.0%; I² = 99.0%; P < 0.001) (**Figure 2A**).

Among patients who completed the full treatment course, CR rates across five trials ranged from 87.5% to 97.0%, yielding a pooled estimate of 90.9% (95% CI: 87.9-93.5%; I² = 55.7%). Corresponding PR rates ranged from 1.0% to 9.9%, pooling to 4.8% (95% CI: 2.4-8.1%; I² = 75.4%). The incidence of SD was uniformly low, ranging from 0% to 1.1%, with a pooled rate of 0.4% (95% CI: 0.0-0.9%; I² = 0.0%). PD occurred in 0-2.0% of patients, yielding a pooled rate of 0.4% (95% CI: 0.0-1.5%; I² = 63.8%) (**Figures 2B–E**).

### 3.4 Pooled safety analysis

**Figure 3:**
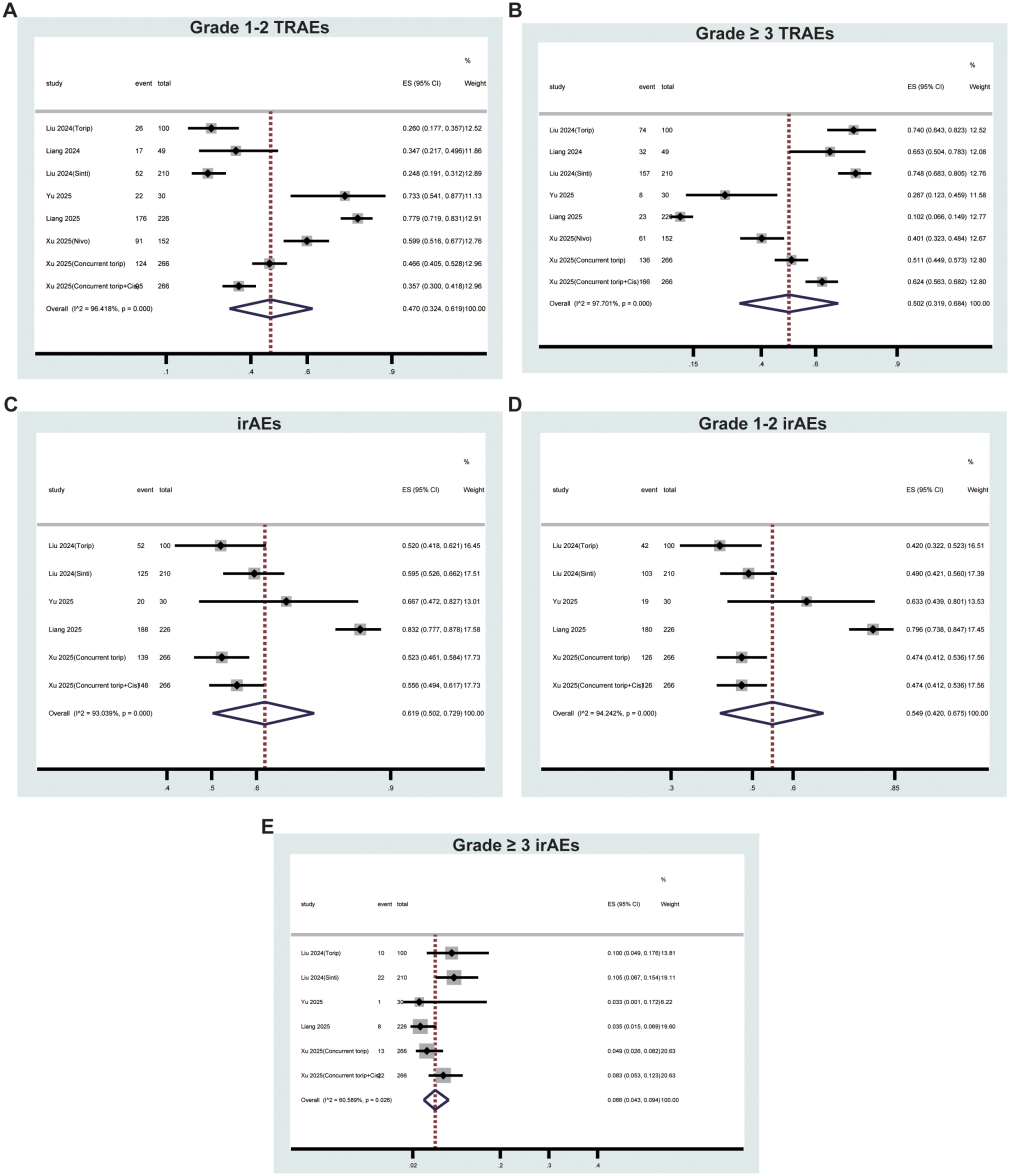
Forest plots of regimen-level and immune-specific safety outcomes in patients with LA-NPC treated with ICI-containing strategies. **(A)** Grade 1–2 TRAEs, **(B)** grade ≥3 TRAEs, **(C)** irAEs, **(D)** grade 1–2 irAEs, and **(E)** grade ≥3 irAEs. LA-NPC, locoregionally advanced nasopharyngeal carcinoma; ICI, Immune checkpoint inhibitor; TRAEs, treatment-related adverse events; irAEs, immune-related adverse events.

Across seven trials, grade 1–2 TRAEs were observed in a pooled 47.0% of patients (95% CI: 32.4-61.9%; I² = 96.4%; P < 0.001) (**Figure 3A****),** while grade ≥3 TRAEs occurred in 50.2% (95% CI: 31.9-68.4%; I² = 97.7%; P < 0.001) (**Figure 3B**). With respect to immune-mediated toxicities, the overall incidence of irAEs was 61.9% (95% CI: 50.2-72.9%; I² = 93.0%; P < 0.001) (**Figure 3C**). Grade 1–2 irAEs were observed in 54.9% of patients (95% CI: 42.0-67.5%; I² = 94.2%; P < 0.001) (**Figure 3D**), while grade ≥3 irAEs occurred in 6.6% of cases (95% CI: 4.3-9.4%; I² = 60.6%; P = 0.026) (**Figure 3E**).

### 3.5 Pooled survival analysis

**Figure 4:**
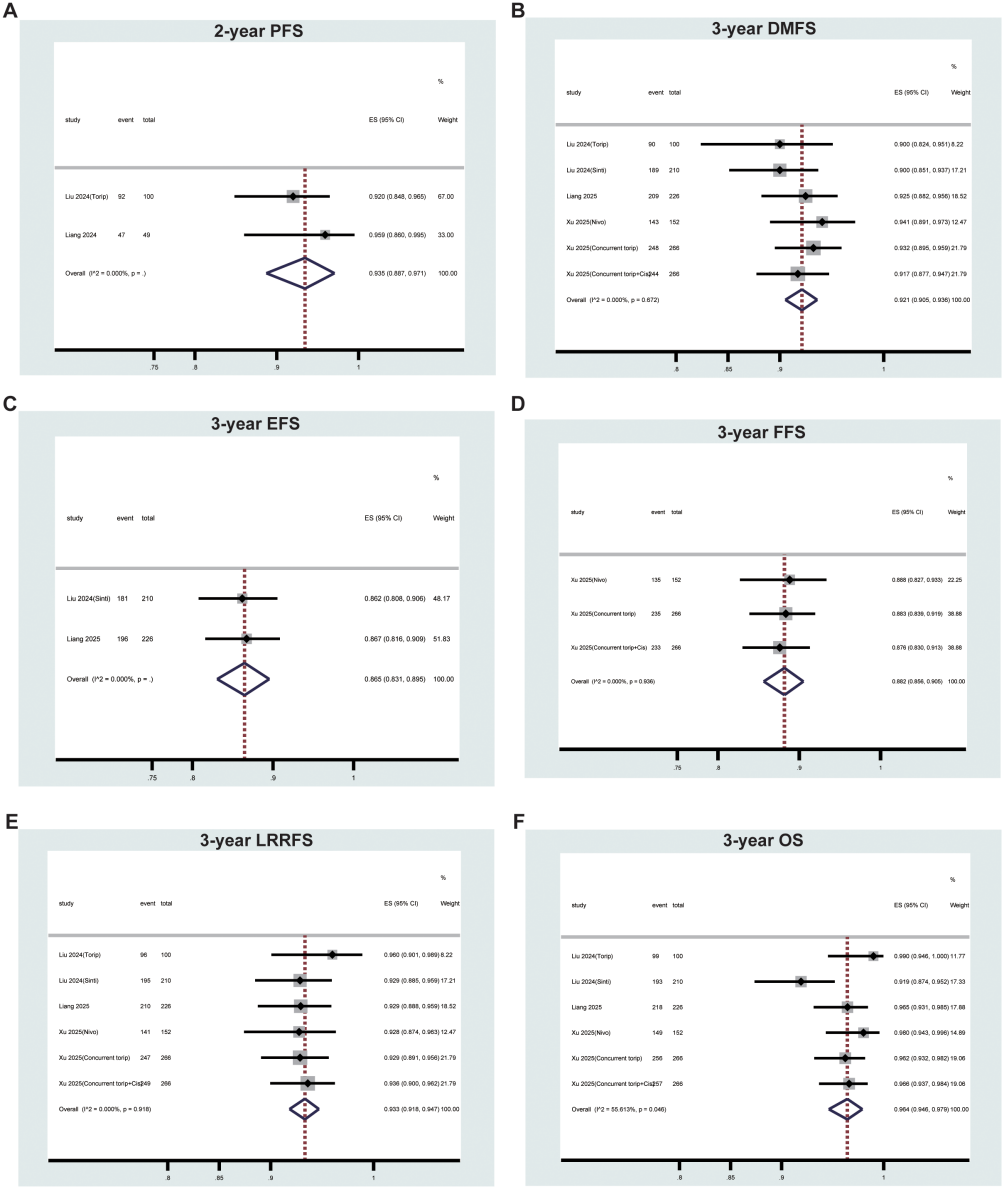
Forest plots of survival outcomes in patients with LA-NPC treated with ICI-containing strategies. **(A)** 2-year PFS, **(B)** 3-year DMFS, **(C)** 3-year EFS, **(D)** 3-year FFS, **(E)** 3-year LRRFS, and **(F)** 3-year OS. LA-NPC, locoregionally advanced nasopharyngeal carcinoma; ICI, Immune checkpoint inhibitor; OS, overall survival; PFS, progression-free survival; LRRFS, locoregional recurrence-free survival; DMFS, distant metastasis-free survival; EFS, event-free survival; FFS, failure-free survival.

Analysis of PFS from two studies (N = 149) yielded a pooled 2-year rate of 93.5% (95% CI: 88.7-97.1%; I² = 0.0%; P < 0.001) (**Figure 4A**). DMFS, evaluated across six studies, produced a pooled 3-year rate of 92.1% (95% CI: 90.5-93.6%; I² = 0.0%; P = 0.672) (**Figure 4B**). The 3-year EFS rate, based on two studies (N = 436), was 86.5% (95% CI: 83.1-89.5%; I² = 0.0%; P < 0.001) (**Figure 4C**). The pooled 3-year FFS across three studies (N = 684) was 88.2% (95% CI: 85.6-90.5%; I² = 0.0%; P = 0.936) (**Figure 4D**). The 3-year LRRFS, derived from six studies, was 93.3% (95% CI: 91.8-94.7%; I² = 0.0%; P = 0.918) (**Figure 4E**). Finally, OS data from six studies involving 1,220 patients were pooled using a random-effects model, yielding a 3-year OS rate of 96.4% (95% CI: 94.6-97.9%; I² = 55.6%; P = 0.046) (**Figure 4F**). Because follow-up remains relatively short in several included trials, these pooled survival estimates should be interpreted as current estimates of early-to-intermediate disease control rather than mature long-term outcomes.

### 3.6 Subgroup analysis

Subgroup analyses were performed to describe outcome patterns across clinically relevant ICI integration strategies rather than to determine the superiority of one strategy over another. Although these analyses were prespecified, several subgroups were informed by a limited number of studies and differed in patient risk profiles, treatment intensity, trial design, and follow-up duration. Because comparisons across strategies were indirect, several endpoints were clinically correlated, and the number of contributing studies and patients varied across subgroups, these findings should be interpreted as exploratory and hypothesis-generating. Interpretation therefore emphasized effect estimates, 95% CIs, between-study heterogeneity, and clinical plausibility, rather than nominal statistical significance alone.

#### 3.6.1 Neoadjuvant treatment regimens

**Figure 5:**
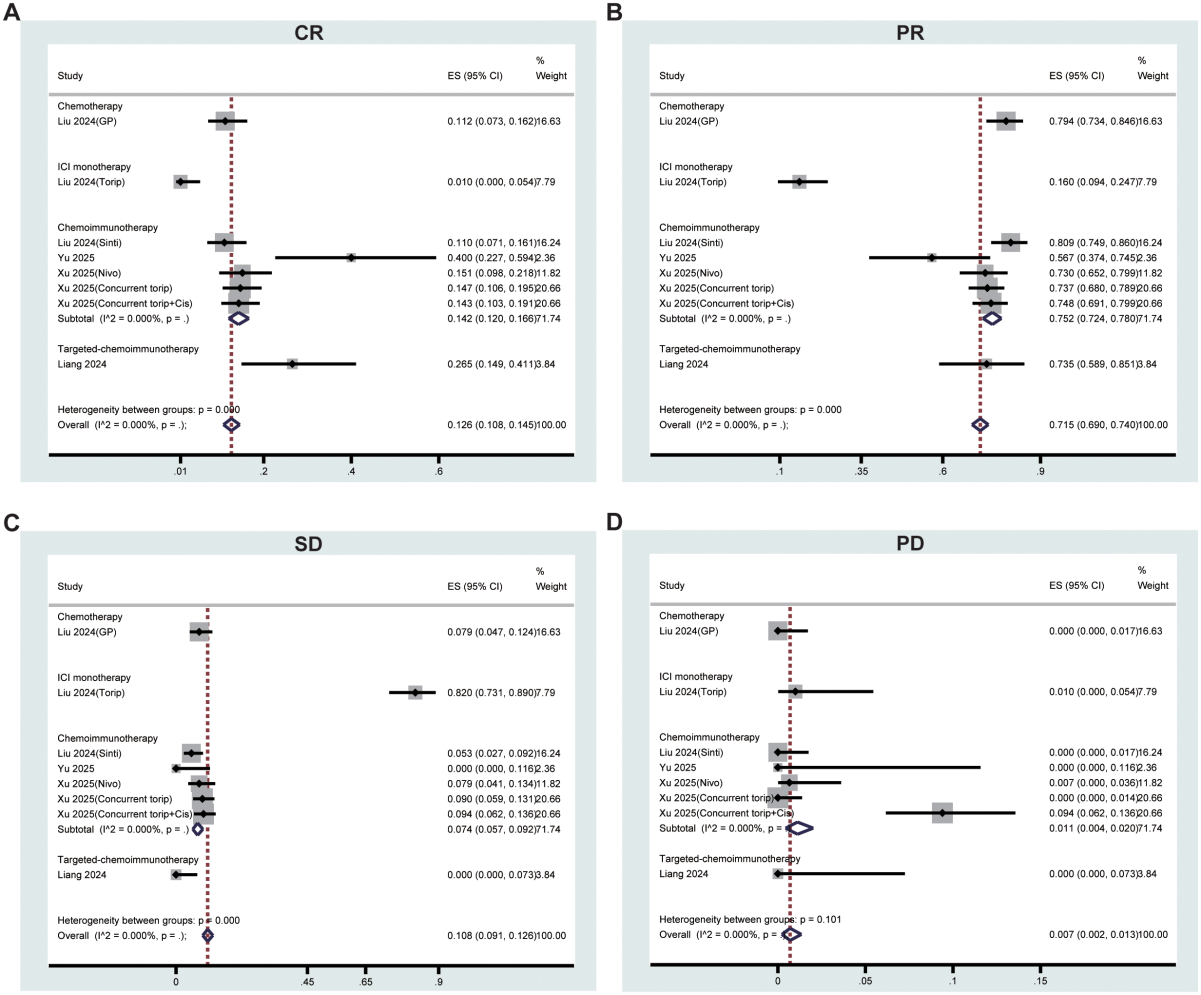
Exploratory subgroup forest plots of tumor activity outcomes after neoadjuvant therapy, stratified by neoadjuvant treatment regimen. **(A)** CR, **(B)** PR, **(C)** SD, and **(D)** PD. LA-NPC, locoregionally advanced nasopharyngeal carcinoma; CR, complete response; PR, partial response; SD, stable disease; PD, progressive disease.

Neoadjuvant strategies were classified into four categories: chemotherapy alone (serving as the reference comparator from two-arm trials), anti-PD-1 monotherapy, chemoimmunotherapy, and chemoimmunotherapy combined with targeted therapy (chemoimmunotherapy-plus-targeted). Following neoadjuvant treatment, CR rates varied markedly across groups: 11.2% (95% CI: 7.3-16.2%) with chemotherapy alone, 1.0% (95% CI: 0.0-5.4%) with anti-PD-1 monotherapy, 14.2% (95% CI: 12.0-16.6%) with chemoimmunotherapy, and 26.5% (95% CI: 14.9-41.1%) with the chemoimmunotherapy-plus-targeted approach (**Figure 5A**). PR rates also differed substantially: 79.4% (95% CI: 73.4-84.6%), 16.0% (95% CI: 9.4-24.7%), 75.2% (95% CI: 72.4-78.0%), and 73.5% (95% CI: 58.9-85.1%) for the four groups, respectively (**Figure 5B**). SD rates were 7.9% (95% CI: 4.7-12.4%) for chemotherapy alone, 82.0% (95% CI: 73.1-89.0%) for anti-PD-1 monotherapy, 7.4% (95% CI: 5.7-9.2%) for chemoimmunotherapy, and 0.0% (95% CI: 0.0-7.3%) for the targeted combination (**Figure 5C**). PD rates remained low across all groups, with no progression events observed in the chemotherapy-alone or chemoimmunotherapy-plus-targeted arms (0.0%; 95% CI: 0.0-1.7% and 0.0-7.3%, respectively); anti-PD-1 monotherapy and chemoimmunotherapy groups showed minimal progression at 1.0% (95% CI: 0.0-5.4%) and 1.1% (95% CI: 0.4-2.0%), respectively (**Figure 5D**).

Upon completion of the full treatment course, CR rates improved substantially across all treatment categories. Chemotherapy alone achieved a CR rate of 92.5% (95% CI: 88.1-95.7%); anti-PD-1 monotherapy achieved 97.0% (95% CI: 91.5-99.4%); chemoimmunotherapy yielded 89.3% (95% CI: 87.2-91.3%); and the chemoimmunotherapy-plus-targeted regimen reached 91.8% (95% CI: 80.4-97.7%) (**Supplementary Figure 3A**). PR rates declined correspondingly to 5.6% (95% CI: 2.9-9.6%), 1.0% (95% CI: 0.0-5.4%), 6.4% (95% CI: 4.9-8.1%), and 2.0% (95% CI: 0.1-10.9%) across the four groups (**Supplementary Figure 3B**). SD incidence was uniformly low at 0.5% (95% CI: 0.0-2.6%), 0.0% (95% CI: 0.0-3.6%), 0.6% (95% CI: 0.1-1.3%), and 0.0% (95% CI: 0.0-7.3%) (**Supplementary Figure 3C**). PD rates were similarly minimal: 0.5% (95% CI: 0.0-2.6%) for chemotherapy alone, 2.0% (95% CI: 0.2-7.0%) for anti-PD-1 monotherapy, 0.2% (95% CI: 0.0-0.6%) for chemoimmunotherapy, and 2.0% (95% CI: 0.1-10.9%) for the chemoimmunotherapy-plus-targeted arm (**Supplementary Figure 3D**).

Safety profiles varied meaningfully across treatment categories. Grade 1–2 TRAEs were reported in 59.2% (95% CI: 54.5-63.7%) of patients receiving chemotherapy alone, 62.7% (95% CI: 57.4-67.9%) with anti-PD-1 monotherapy, 41.2% (95% CI: 38.1-44.5%) with chemoimmunotherapy, and 34.7% (95% CI: 21.7-49.6%) with the targeted combination (**Supplementary Figure 4A**). Grade ≥3 TRAE rates were markedly higher in the chemoimmunotherapy and targeted combination groups at 57.4% (95% CI: 54.2-60.6%) and 65.3% (95% CI: 50.4-78.3%), respectively, compared with 27.5% (95% CI: 23.4-31.8%) for chemotherapy alone and 26.7% (95% CI: 22.0-31.6%) for anti-PD-1 monotherapy (**Supplementary Figure 4B**).

Regarding long-term outcomes, 3-year DMFS was lowest with chemotherapy alone at 83.6% (95% CI: 80.0-86.9%), while anti-PD-1 monotherapy and chemoimmunotherapy achieved higher rates of 91.8% (95% CI: 88.5-94.6%) and 92.2% (95% CI: 90.4-93.9%), respectively (**Supplementary Figure 4C**). The 3-year EFS followed a similar pattern: 76.5% (95% CI: 72.4-80.4%) for chemotherapy alone, versus 86.7% (95% CI: 81.6-90.9%) for anti-PD-1 monotherapy and 86.2% (95% CI: 80.8-90.6%) for chemoimmunotherapy (**Supplementary Figure 4D**). LRRFS at 3 years was 86.6% (95% CI: 83.2-89.6%) for chemotherapy alone, 94.0% (95% CI: 91.1-96.4%) for anti-PD-1 monotherapy, and 93.1% (95% CI: 91.3-94.7%) for chemoimmunotherapy (**Supplementary Figure 4E**). Pooled 3-year OS rates were 92.5% (95% CI: 89.8-94.8%) for chemotherapy alone, 97.4% (95% CI: 95.3-98.9%) for anti-PD-1 monotherapy, and 95.9% (95% CI: 94.4-97.1%) for chemoimmunotherapy (**Supplementary Figure 4F**).

#### 3.6.2 Neoadjuvant chemotherapy regimens

For patients undergoing neoadjuvant chemoimmunotherapy, the chemotherapy backbone fell predominantly into two categories: the GP doublet (gemcitabine plus cisplatin), used in three studies, and a modified TPF regimen (nab-paclitaxel with either cisplatin-capecitabine or cisplatin-S-1), employed in two studies. Exploratory subgroup estimates suggested different tumor activity and safety patterns between these chemotherapy backbones.

**Figure 6:**
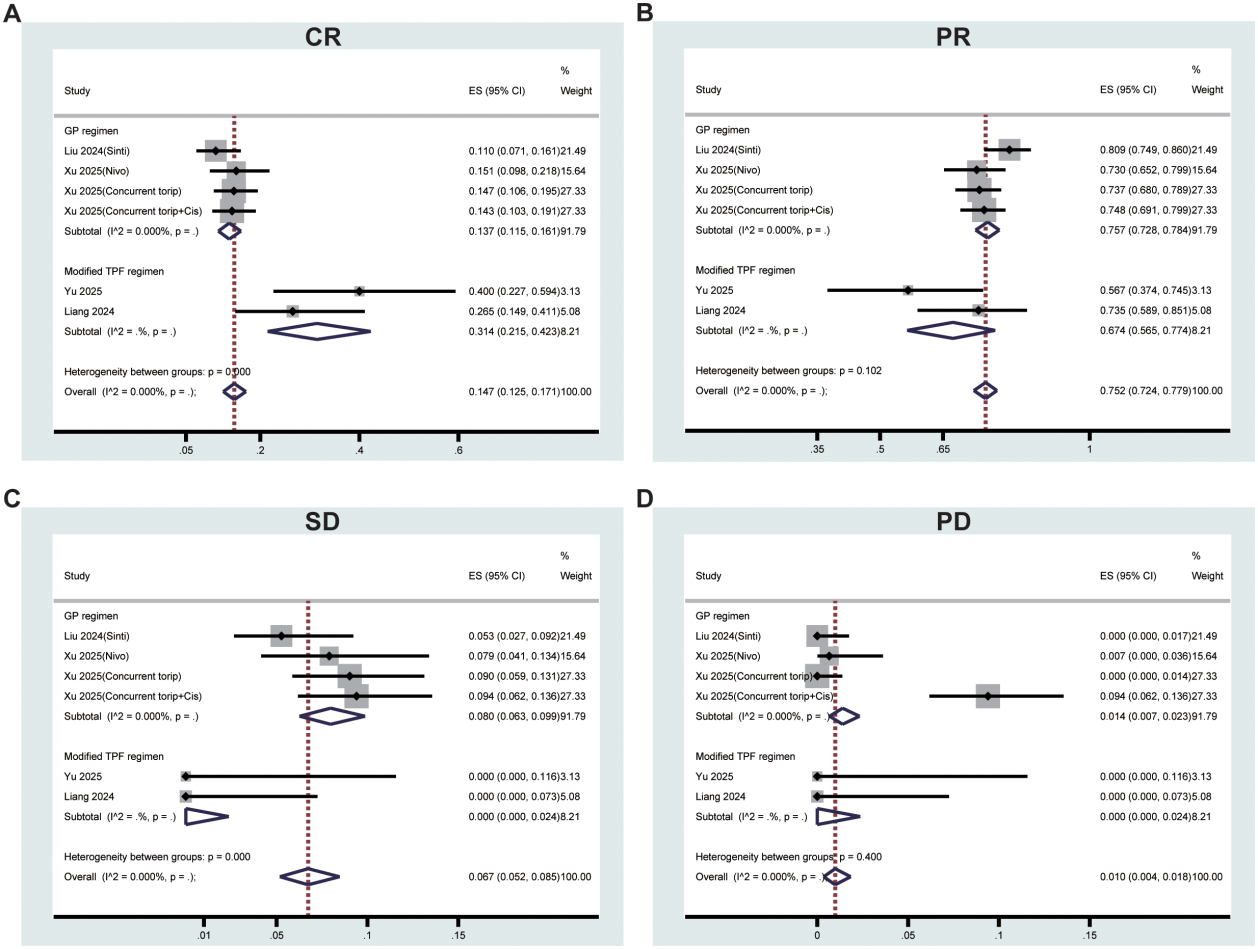
Exploratory subgroup forest plots of tumor activity outcomes after neoadjuvant therapy, stratified by neoadjuvant chemotherapy backbone. **(A)** CR, **(B)** PR, **(C)** SD, and **(D)** PD. LA-NPC, locoregionally advanced nasopharyngeal carcinoma; CR, complete response; PR, partial response; SD, stable disease; PD, progressive disease.

Following neoadjuvant treatment, the CR rate was substantially higher in the TPF group at 31.4% (95% CI: 21.5-42.3%) compared with 13.7% (95% CI: 11.5-16.1%) in the GP group (**Figure 6A**). PR rates were 67.4% (95% CI: 56.5-77.4%) for TPF and 75.7% (95% CI: 72.8-78.4%) for GP (**Figure 6B**). SD was observed in 8.0% (95% CI: 6.3-9.9%) of GP-treated patients, while no SD was recorded in the TPF group (95% CI: 0.0-2.4%) (**Figure 6C**). PD occurred in 1.4% (95% CI: 0.7-2.3%) of patients in the GP group, with no PD events observed with TPF (95% CI: 0.0-2.4%) (**Figure 6D**).

After completion of the full treatment course, CR rates converged between arms: 89.3% (95% CI: 87.2-91.3%) for GP and 91.8% (95% CI: 80.4-97.7%) for TPF; PR rates were 6.4% (95% CI: 4.9-8.1%) and 2.0% (95% CI: 0.1-10.9%), respectively. SD remained minimal in both groups (GP: 0.6% [95% CI: 0.1-1.3%]; TPF: 0.0% [95% CI: 0.0-7.3%]), as did PD rates (GP: 0.2% [95% CI: 0.0-0.6%]; TPF: 2.0% [95% CI: 0.1-10.9%]) (**Supplementary Figures 5A–D**).

Safety profiles differed between the two backbones. Grade 1–2 TRAEs were reported in 40.2% (95% CI: 37.0-43.5%) of GP patients and 49.5% (95% CI: 38.4-60.7%) of TPF patients (**Supplementary Figure 5E**). Grade ≥3 TRAEs were more frequent with GP (58.4%; 95% CI: 55.2-61.6%) than with TPF (50.5%; 95% CI: 39.3-61.6%) (**Supplementary Figure 5F**).

#### 3.6.3 Immunotherapy agents

Agent-level subgroup analyses were performed descriptively because individual ICIs were evaluated in different treatment contexts and were not compared directly within the same randomized framework. Camrelizumab was evaluated in three of the seven included studies. In the neoadjuvant setting, camrelizumab-based regimens were associated with a pooled CR rate of 31.4% (95% CI: 21.5-42.3%) and a PR rate of 67.4% (95% CI: 56.5-77.4%) (**Supplementary Figures 6A, B**). Notably, neither SD nor PD events were recorded in these neoadjuvant cohorts (both 0.0%; 95% CI: 0.0-2.4%) (**Supplementary Figures 6C, D**). Among patients completing the full treatment course, ORR was 100% (**Supplementary Figure 6E**). The safety profile of camrelizumab showed grade 1–2 TRAEs in 63.0% (95% CI: 34.0-88.0%) and grade ≥3 TRAEs in 31.8% (95% CI: 3.4-71.0%) of patients (**Supplementary Figures 7A, B**). IrAEs were common, with an overall incidence of 81.8% (95% CI: 76.7-86.4%) (**Supplementary Figure 7C**); grade 1–2 irAEs accounted for 78.2% (95% CI: 72.9-83.2%) and grade ≥3 irAEs for 3.0% (95% CI: 1.1-5.8%) (**Supplementary Figures 7D, E**).

Toripalimab, evaluated in three trials, demonstrated a neoadjuvant CR rate of 8.9% (95% CI: 2.4-18.8%) and a PR rate of 54.8% (95% CI: 23.4-84.2%) (**Supplementary Figures 8A, B**). Among patients completing the full treatment course, the CR rate rose to 91.4% (95% CI: 85.9-95.6%), with a corresponding PR rate of 5.2% (95% CI: 1.8-9.9%) (**Supplementary Figures 8C, D**). The safety profile of toripalimab was comparatively favorable: grade 1–2 TRAEs occurred in 36.4% (95% CI: 26.1-47.5%) and grade ≥3 TRAEs in 62.2% (95% CI: 50.2-73.6%) of patients (**Supplementary Figures 9A, B**). The overall irAE rate was 53.6% (95% CI: 49.7-57.5%) (**Supplementary Figure 9C**), comprising grade 1–2 irAEs in 46.5% (95% CI: 42.6-50.4%) and grade ≥3 irAEs in 7.2% (95% CI: 4.5-10.5%) (**Supplementary Figures 9D, E**). Toripalimab-based regimens showed high long-term survival estimates: 3-year DMFS 92.2% (95% CI: 89.9-94.2%) (**Supplementary Figure 10A**), 3-year FFS 88.0% (95% CI: 85.1-90.6%) (**Supplementary Figure 10B**), 3-year LRRFS 93.8% (95% CI: 91.7-95.6%) (**Supplementary Figure 10C**), and 3-year OS 97.0% (95% CI: 95.4-98.2%) (**Supplementary Figure 10D**).

#### 3.6.4 Immunotherapy timing

**Figure 7:**
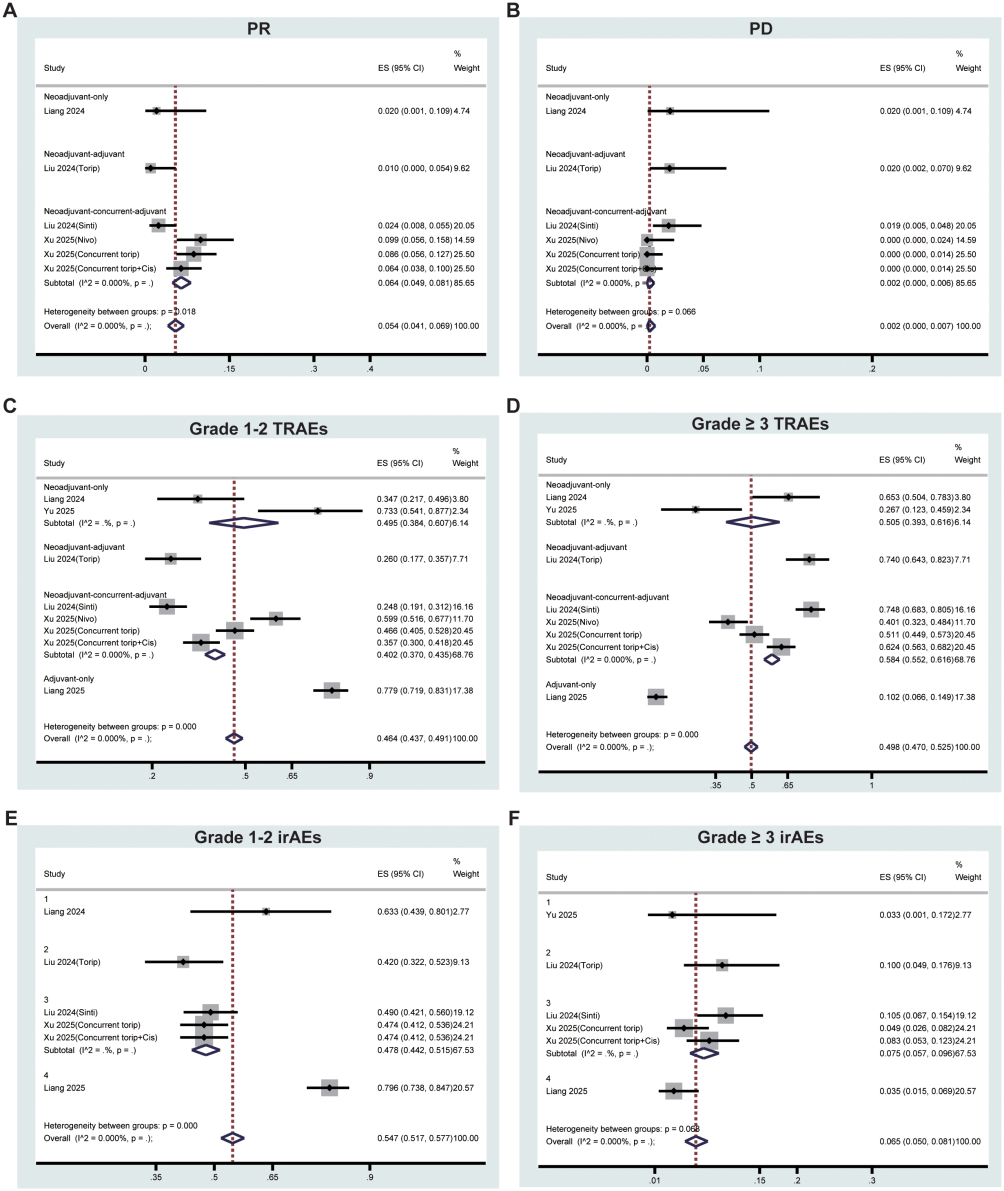
Exploratory subgroup forest plots of tumor activity and safety outcomes after completion of the full treatment regimen, stratified by ICI timing. **(A)** PR, **(B)** PD, **(C)** grade 1–2 TRAEs, **(D)** grade ≥3 TRAEs, **(E)** grade 1–2 irAEs, and **(F)** grade ≥3 irAEs. LA-NPC, locoregionally advanced nasopharyngeal carcinoma; PR, partial response; PD, progressive disease; TRAEs, treatment-related adverse events; irAEs, immune-related adverse events.

Subgroup analysis stratified by the timing of ICI administration showed different tumor activity and safety patterns across the four sequencing strategies evaluated. Among patients who completed the full treatment course, the neoadjuvant-concurrent-adjuvant strategy was associated with the highest PR rate (6.4%; 95% CI: 4.9-8.1%) and the lowest rate of PD (0.2%; 95% CI: 0.0-0.6%), compared with neoadjuvant-only, neoadjuvant-adjuvant, and adjuvant-only approaches (**Figures 7A, B**).

Regarding treatment-related toxicity, adjuvant-only therapy was associated with the highest frequency of grade 1–2 TRAEs (77.9%; 95% CI: 71.9-83.1%) (**Figure 7C**), while the neoadjuvant-adjuvant regimen exhibited the highest rate of grade ≥3 TRAEs (74.0%; 95% CI: 64.3-82.3%) (**Figure 7D**). Grade 1–2 irAEs were most frequent with adjuvant-only therapy (79.6%; 95% CI: 73.8-84.7%), followed by neoadjuvant-only (63.3%; 95% CI: 43.9-80.1%), neoadjuvant-concurrent-adjuvant (47.8%; 95% CI: 44.2-51.5%), and neoadjuvant-adjuvant (42.0%; 95% CI: 32.2-52.3%) strategies (**Figure 7E**). Grade ≥3 irAEs occurred most frequently with the neoadjuvant-adjuvant regimen (10.0%; 95% CI: 4.9-17.6%), followed by neoadjuvant-concurrent-adjuvant (7.5%; 95% CI: 5.7-9.6%), adjuvant-only (3.5%; 95% CI: 1.5-6.9%), and neoadjuvant-only approaches (3.3%; 95% CI: 0.1-17.2%) (**Figure 7F**).

**Figure 8:**
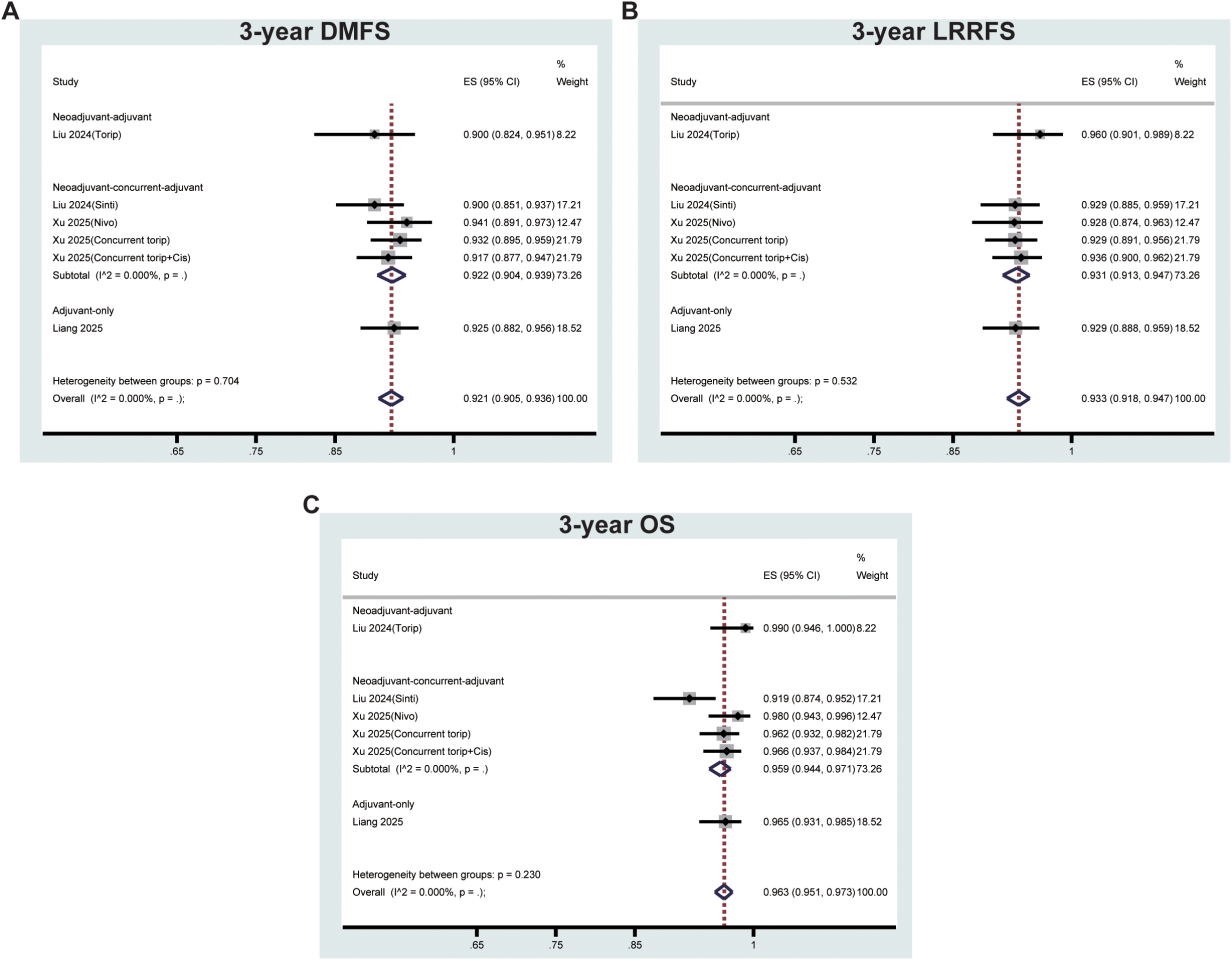
Exploratory subgroup forest plots of survival outcomes after completion of the full treatment regimen, stratified by ICI timing. **(A)** 3-year DMFS, **(B)** 3-year LRRFS, and **(C)** 3-year OS. LA-NPC, locoregionally advanced nasopharyngeal carcinoma; OS, overall survival; LRRFS, locoregional recurrence-free survival; DMFS distant metastasis-free survival.

In terms of long-term outcomes, 3-year DMFS estimates were numerically similar across the neoadjuvant-adjuvant, neoadjuvant-concurrent-adjuvant, and adjuvant-only strategies, at 90.0% (95% CI: 82.4-95.1%), 92.2% (95% CI: 90.4-93.9%), and 92.5% (95% CI: 88.2-95.6%), respectively (**Figure 8A**). The neoadjuvant-adjuvant regimen showed numerically higher 3-year LRRFS and OS estimates, at 96.0% (95% CI: 90.1-98.9%) and 99.0% (95% CI: 94.6-100.0%), respectively (**Figures 8B, C**). These differences should be interpreted descriptively because comparisons across timing strategies were indirect and not adjusted for multiplicity.

### 3.7 Sensitivity analysis and publication bias

**Figure 9:**
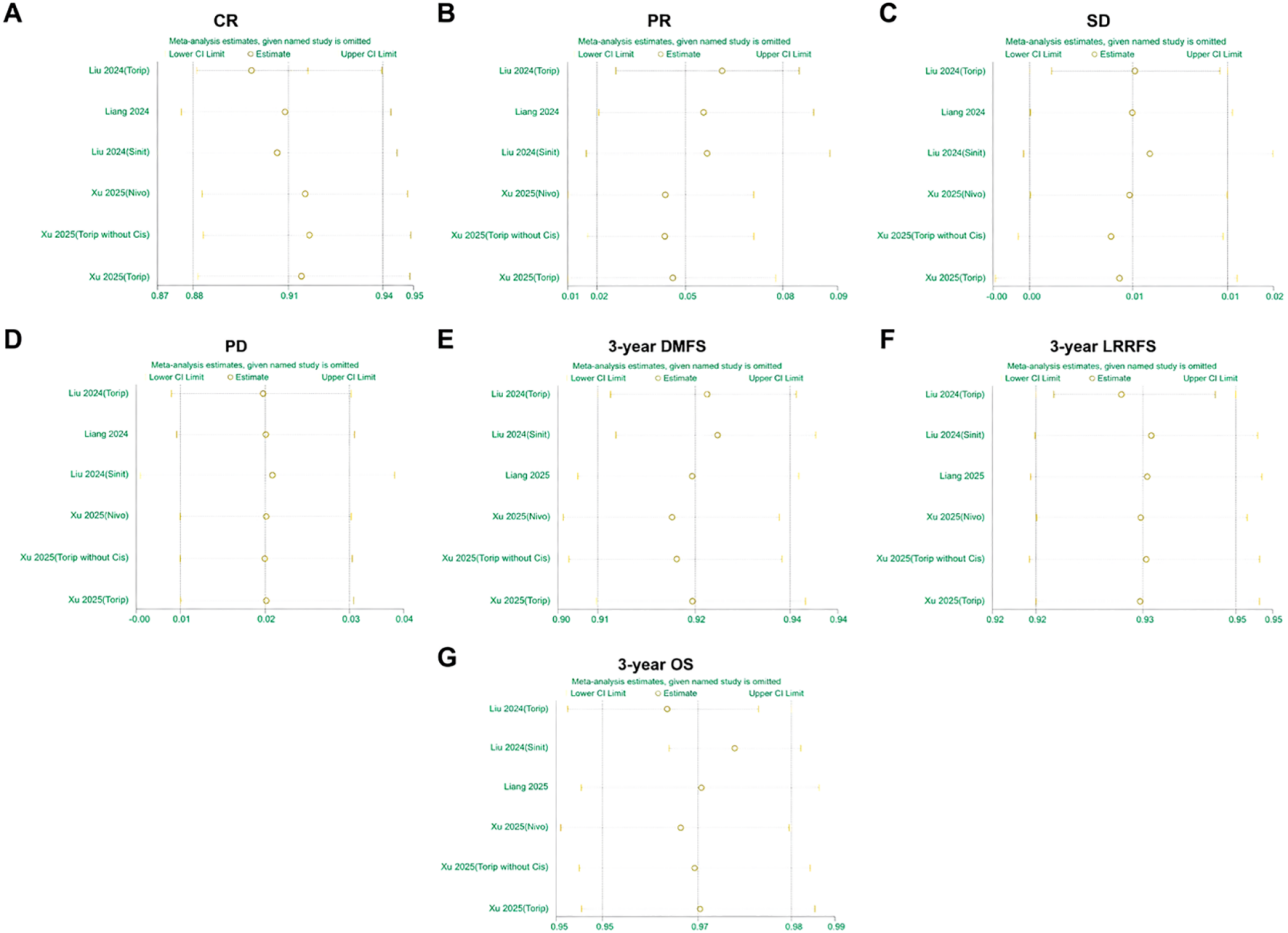
Sensitivity analyses of pooled tumor activity and survival estimates in patients with LA-NPC treated with ICI-containing strategies. **(A)** CR, **(B)** PR, **(C)** SD, and **(D)** PD after completion of the full treatment regimen; **(E)** 3-year DMFS, **(F)** 3-year LRRFS, and **(G)** 3-year OS. LA-NPC, locoregionally advanced nasopharyngeal carcinoma; CR, complete response; PR, partial response; SD, stable disease; PD, progressive disease; OS, overall survival; LRRFS, locoregional recurrence-free survival; DMFS, distant metastasis-free survival.

**Figure 10:**
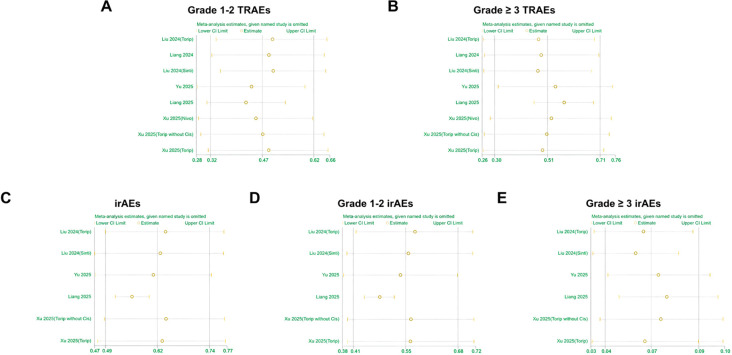
Sensitivity analyses of pooled regimen-level and immune-specific safety estimates in patients with LA-NPC treated with ICI-containing strategies. **(A)** Grade 1–2 TRAEs, **(B)** grade ≥3 TRAEs, **(C)** irAEs, **(D)** grade 1–2 irAEs, and **(E)** grade ≥3 irAEs. LA-NPC, locoregionally advanced nasopharyngeal carcinoma; ICI, Immune checkpoint inhibitor; TRAEs, treatment-related adverse events; irAEs, immune-related adverse events.

To assess the robustness of pooled estimates in the context of observed heterogeneity, a sensitivity analysis was performed by sequentially omitting each individual study and recalculating the remaining pooled estimates. This approach confirmed that neither efficacy outcomes (**Figures 9A–G**; **Supplementary Figures 11A–G**) nor safety endpoints (**Figures 10A–E**) were materially altered by the exclusion of any single trial, supporting the overall stability and reliability of our findings.

Funnel plot analyses combined with Egger’s regression tests revealed no evidence of substantial publication bias across multiple prespecified endpoints. For tumor activity endpoints, no significant asymmetry was detected for ORR after neoadjuvant therapy (P = 0.265) or for CR (P = 0.126), PR (P = 0.808), SD (P = 0.061), or PD (P = 0.069) after completion of the full treatment course (**Supplementary Figures 12A–E**). Similarly, no significant publication bias was identified for grade ≥3 irAEs (P = 0.079), 3-year FFS (P = 0.064), or 3-year OS (P = 0.789) (**Supplementary Figure 12F–H**).

## 4 Discussion

In this systematic review and meta-analysis of prospective studies, ICI-containing strategies for LA-NPC showed favorable current survival estimates, measurable tumor activity, and generally manageable toxicity. These findings should be interpreted in the context of recent randomized trials supporting the integration of ICIs into definitive treatment (22–24, 29). Accordingly, the main contribution of this study is not to re-examine whether ICIs have activity in LA-NPC, but to summarize how different ICI-containing strategies have been evaluated across clinically relevant treatment settings. By considering treatment modality, timing of ICI administration, chemotherapy backbone, treatment duration, and individual ICI agent, this review describes the observed survival estimates, tumor activity, and toxicity trade-offs reported in contemporary prospective studies (21–24, 29–31). However, because several subgroup comparisons were based on a limited number of heterogeneous studies with relatively short follow-up, these findings should be interpreted as descriptive and hypothesis-generating rather than as evidence of comparative superiority.

The numerically highest induction-phase CR estimate was observed with chemoimmunotherapy combined with antiangiogenic therapy (21). This finding is biologically plausible, because antiangiogenic agents may normalize tumor vasculature, reduce hypoxia, and reshape the immune microenvironment in ways that could enhance checkpoint blockade (32–35). However, this result should be interpreted with caution. The apparent advantage may also reflect the greater cytotoxic intensity of the modified taxane-platinum-fluoropyrimidine backbone, differences in baseline risk across enrolled populations, and the small number of contributing studies. The higher grade 3 or higher adverse event estimate further suggests that intensified induction strategies are unlikely to be appropriate for all patients. These findings support further prospective evaluation of intensified chemoimmunotherapy-based regimens in carefully selected high-risk patients, with close attention to treatment completion, toxicity management, quality-of-life outcomes, and the durability of response during longer follow-up.

Treatment sequencing was another important source of heterogeneity. Full-course ICI schedules that incorporated immunotherapy during induction, concurrent, and adjuvant phases were associated with the lowest pooled PD estimate, whereas induction-adjuvant schedules showed numerically favorable LRRFS and OS estimates (23, 29, 31). These findings should not be read as evidence that one schedule is superior to another, because the comparisons were indirect and differed in trial design, chemotherapy intensity, patient risk profile, and follow-up duration. Still, they underscore a practical treatment trade-off. Broader ICI exposure may be associated with higher disease-control estimates but can increase cumulative toxicity, whereas selected phase-restricted or cisplatin-sparing strategies may preserve tolerability and quality of life. In particular, DIAMOND showed that a toripalimab-based strategy without concurrent cisplatin may maintain disease control while reducing treatment burden in selected patients (24). Future trials should prospectively define the timing and duration of ICI therapy and incorporate patient-reported outcomes, Epstein-Barr virus DNA kinetics, biomarker-defined risk stratification, and sufficiently mature follow-up to assess the durability of survival benefit.

The chemotherapy backbone also appeared to influence early tumor response. In the induction phase, modified taxane-platinum-fluoropyrimidine regimens were associated with a higher CR estimate than gemcitabine-cisplatin-based regimens, although this was an indirect comparison across a small number of studies (21, 23, 24, 30, 31). This difference should not be attributed to the chemotherapy backbone alone. It may also reflect differences in cytotoxic intensity, taxane-containing triplet components, patient selection, disease risk, and treatment context. Preclinical and translational studies suggest that taxanes can modulate antigen presentation, tumor-associated macrophages, and other immune features that may interact with checkpoint blockade, but these mechanisms remain inferential in LA-NPC (36–38). These findings support further prospective evaluation of chemotherapy backbones in ICI-containing induction regimens rather than defining one backbone as preferred, particularly because the available comparisons were indirect and based on a limited number of heterogeneous studies.

Agent-level subgroup estimates also varied, but these findings require cautious interpretation because individual ICIs were embedded within different treatment regimens and trial contexts. Camrelizumab-based regimens showed a high neoadjuvant response estimate in studies that also incorporated intensified chemotherapy and/or antiangiogenic therapy, whereas toripalimab-based regimens yielded high survival estimates with a moderate incidence of irAEs (21–24, 29–31). These descriptive findings suggest that agent-specific and regimen-specific profiles may be relevant when designing future trials, but they do not establish the superiority of one ICI over another. Prospective studies with standardized treatment backbones, biomarker-defined stratification, and longer follow-up are needed to clarify whether observed differences are attributable to the ICI agent itself, the accompanying treatment regimen, patient selection, or differences in follow-up maturity.

Several limitations of this meta-analysis warrant consideration. First, heterogeneity in patient risk stratification across trials, including cohorts enriched for N3-stage disease (21) and trials enrolling predominantly stage III populations (29), may have influenced pooled estimates and subgroup comparisons. Second, the inclusion of non-randomized and single-arm prospective studies (21, 30, 31) may have overestimated treatment effects, although leave-one-out sensitivity analyses did not suggest that any single study drove the overall findings. Third, several prespecified subgroup analyses were based on a limited number of studies, and the included populations differed in disease stage, risk profile, treatment intensity, ICI timing, chemotherapy backbone, trial design, and follow-up duration. Therefore, subgroup findings should be interpreted as descriptive and hypothesis-generating rather than as direct comparisons between treatment strategies. Fourth, survival data remain relatively immature in several included trials, with median follow-up durations ranging from 24 to 37 months; longer follow-up is needed to determine whether the currently favorable OS, FFS, DMFS, and locoregional control estimates are maintained and to better characterize late toxicity after intensified ICI-containing regimens. Fifth, the large number of outcomes and subgroup analyses requires caution. Response categories, survival outcomes, toxicity measures, and treatment-strategy subgroups were closely related clinically, and these analyses were not designed as independent confirmatory tests. We did not apply formal multiplicity correction because this review was intended to summarize patterns across prospective studies rather than establish statistical superiority among ICI strategies. Therefore, nominal differences, particularly in subgroup analyses, should be interpreted as exploratory and hypothesis-generating. Finally, all included trials enrolled patients from Chinese institutions (21–24, 29–31), which may limit generalizability to non-Asian populations given the geographic and molecular heterogeneity of NPC (1, 4). Future studies should prospectively evaluate optimized ICI timing, treatment duration, chemotherapy backbone, biomarker-defined patient selection, late toxicity, and patient-reported outcomes with longer follow-up.

## 5 Conclusion

In conclusion, this systematic review and meta-analysis of prospective studies suggests that, at the currently available follow-up, ICI-containing strategies are associated with favorable survival estimates, measurable tumor activity, and acceptable toxicity in LA-NPC. Outcomes appeared to vary according to treatment composition, timing of ICI use, chemotherapy backbone, duration of ICI exposure, and ICI agent. However, because these comparisons were indirect, involved clinically correlated outcomes, included heterogeneous populations, and were not adjusted for multiplicity, they should be interpreted as exploratory. Longer follow-up is also required to confirm the durability of survival benefit and to better define late toxicity. Rather than defining a single preferred ICI strategy, this study provides a framework for optimizing ICI integration and designing future randomized, biomarker-informed trials.

## Data Availability

The original contributions presented in the study are included in the article/**Supplementary Material**. Further inquiries can be directed to the corresponding authors.

## Supplementary material

Supplementary Figure 1 Quality assessment of the included studies. **(A)** Risk-of-bias assessment of randomized controlled trials using the Cochrane Collaboration tool; **(B)** risk-of-bias assessment of non-randomized and single-arm studies using ROBINS-I. RCTs, randomized controlled trials; ROBINS-I, the Risk Of Bias In Non-randomized Studies of Interventions.

Supplementary Figure 2 Forest plots of tumor activity outcomes after neoadjuvant ICI-containing therapy. **(A)** CR, **(B)** PR, **(C)** SD, and **(D)** PD. LA-NPC, locoregionally advanced nasopharyngeal carcinoma; ICI, Immune checkpoint inhibitor; CR, complete response; PR, partial response; SD, stable disease; PD, progressive disease.

Supplementary Figure 3 Exploratory subgroup forest plots of tumor activity outcomes after completion of the full treatment regimen, stratified by neoadjuvant treatment regimen. **(A)** CR, **(B)** PR, **(C)** SD, and **(D)** PD. LA-NPC, locoregionally advanced nasopharyngeal carcinoma; CR, complete response; PR, partial response; SD, stable disease; PD, progressive disease.

Supplementary Figure 4 Exploratory subgroup forest plots of safety and survival outcomes, stratified by neoadjuvant treatment regimen. **(A)** Grade 1–2 TRAEs, **(B)** grade ≥3 TRAEs, **(C)** 3-year DMFS, **(D)** 3-year EFS, **(E)** 3-year LRRFS, and **(F)** 3-year OS. LA-NPC, locoregionally advanced nasopharyngeal carcinoma; TRAEs, treatment-related adverse events; OS, overall survival; LRRFS, locoregional recurrence-free survival; DMFS, distant metastasis-free survival; EFS, event-free survival.

Supplementary Figure 5 Exploratory subgroup forest plots of tumor activity and safety outcomes after completion of the full treatment regimen, stratified by neoadjuvant chemotherapy backbone. **(A)** CR, **(B)** PR, **(C)** SD, **(D)** PD, **(E)** grade 1–2 TRAEs, and **(F)** grade ≥3 TRAEs. LA-NPC, locoregionally advanced nasopharyngeal carcinoma; CR, complete response; PR, partial response; SD, stable disease; PD, progressive disease.

Supplementary Figure 6 Forest plots of tumor activity outcomes in patients with LA-NPC treated with camrelizumab-containing strategies. **(A)** CR, **(B)** PR, **(C)** SD, and **(D)** PD after neoadjuvant ICI-containing therapy; **(E)** ORR after completion of the full treatment regimen. LA-NPC, locoregionally advanced nasopharyngeal carcinoma; CR, complete response; PR, partial response; SD, stable disease; PD, progressive disease; ORR, objective response rate.

Supplementary Figure 7 Forest plots of regimen-level and immune-specific safety outcomes in patients with LA-NPC treated with camrelizumab-containing strategies. **(A)** Grade 1–2 TRAEs, **(B)** grade ≥3 TRAEs, **(C)** irAEs, **(D)** grade 1–2 irAEs, and **(E)** grade ≥3 irAEs. LA-NPC, locoregionally advanced nasopharyngeal carcinoma; TRAEs, treatment-related adverse events; irAEs, immune-related adverse events.

Supplementary Figure 8 Forest plots of tumor activity outcomes in patients with LA-NPC treated with toripalimab-containing strategies. **(A)** CR and **(B)** PR after neoadjuvant ICI-containing therapy; **(C)** CR and **(D)** PR after completion of the full treatment regimen. LA-NPC, locoregionally advanced nasopharyngeal carcinoma; CR, complete response; PR, partial response.

Supplementary Figure 9 Forest plots of regimen-level and immune-specific safety outcomes in patients with LA-NPC treated with toripalimab-containing strategies. **(A)** Grade 1–2 TRAEs, **(B)** grade ≥3 TRAEs, **(C)** irAEs, **(D)** grade 1–2 irAEs, and **(E)** grade ≥3 irAEs. LA-NPC, locoregionally advanced nasopharyngeal carcinoma; TRAEs, treatment-related adverse events; irAEs, immune-related adverse events.

Supplementary Figure 10 Forest plots of survival outcomes in patients with LA-NPC treated with toripalimab-containing strategies. **(A)** 3-year DMFS, **(B)** 3-year FFS, **(C)** 3-year LRRFS, and **(D)** 3-year OS. LA-NPC, locoregionally advanced nasopharyngeal carcinoma; OS, overall survival; LRRFS, locoregional recurrence-free survival; DMFS, distant metastasis-free survival; FFS, failure-free survival.

Supplementary Figure 11 Sensitivity analyses of pooled tumor activity and survival estimates in patients with LA-NPC treated with ICI-containing strategies. **(A)** CR, **(B)** PR, **(C)** SD, and **(D)** PD after neoadjuvant therapy; **(E)** 2-year PFS, **(F)** 3-year EFS, and **(G)** 3-year FFS. LA-NPC, locoregionally advanced nasopharyngeal carcinoma; CR, complete response; PR, partial response; SD, stable disease; PD, progressive disease; PFS, progression-free survival; EFS, event-free survival; FFS, failure-free survival.

Supplementary Figure 12 Egger’s tests for small-study effects in pooled estimates. **(A)** ORR after neoadjuvant therapy, **(B)** CR, **(C)** PR, **(D)** SD, and **(E)** PD after completion of the full treatment regimen; **(F)** grade ≥3 irAEs, **(G)** 3-year FFS, and **(H)** 3-year OS. CR, complete response; PR, partial response; SD, stable disease; PD, progressive disease; irAEs, immune-related adverse events; OS, overall survival; FFS, failure-free survival.

Supplementary Table 1 Detailed search strategy used in this systematic review and meta-analysis.
