# Supplementary figures and images for: Immune checkpoint inhibitor integration in locoregionally advanced nasopharyngeal carcinoma: a prospective evidence synthesis on efficacy, safety, and therapeutic optimization
Source: Front Immunol. 2026 Jun 26;17:1840292. doi: 10.3389/fimmu.2026.1840292 (PMC13350252; doi:10.3389/fimmu.2026.1840292)

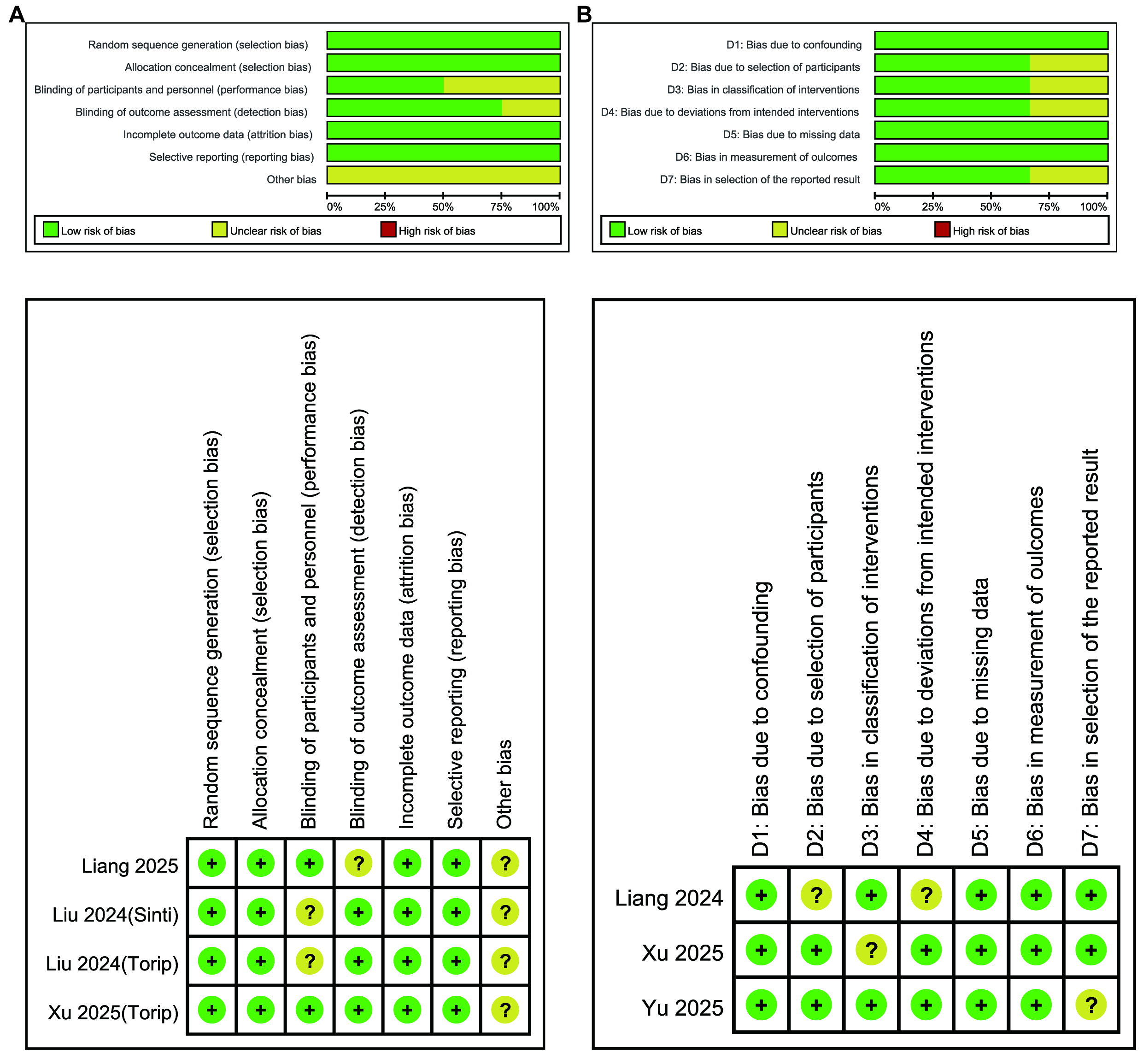

Supplement: Supplementary Figure 1 — Quality assessment of the included studies. (A) Risk-of-bias assessment of randomized controlled trials using the Cochrane Collaboration tool; (B) risk-of-bias assessment of non-randomized and single-arm studies using ROBINS-I. RCTs, randomized controlled trials; ROBINS-I, the Risk Of Bias In Non-randomized Studies of Interventions. [file Image1.tif]

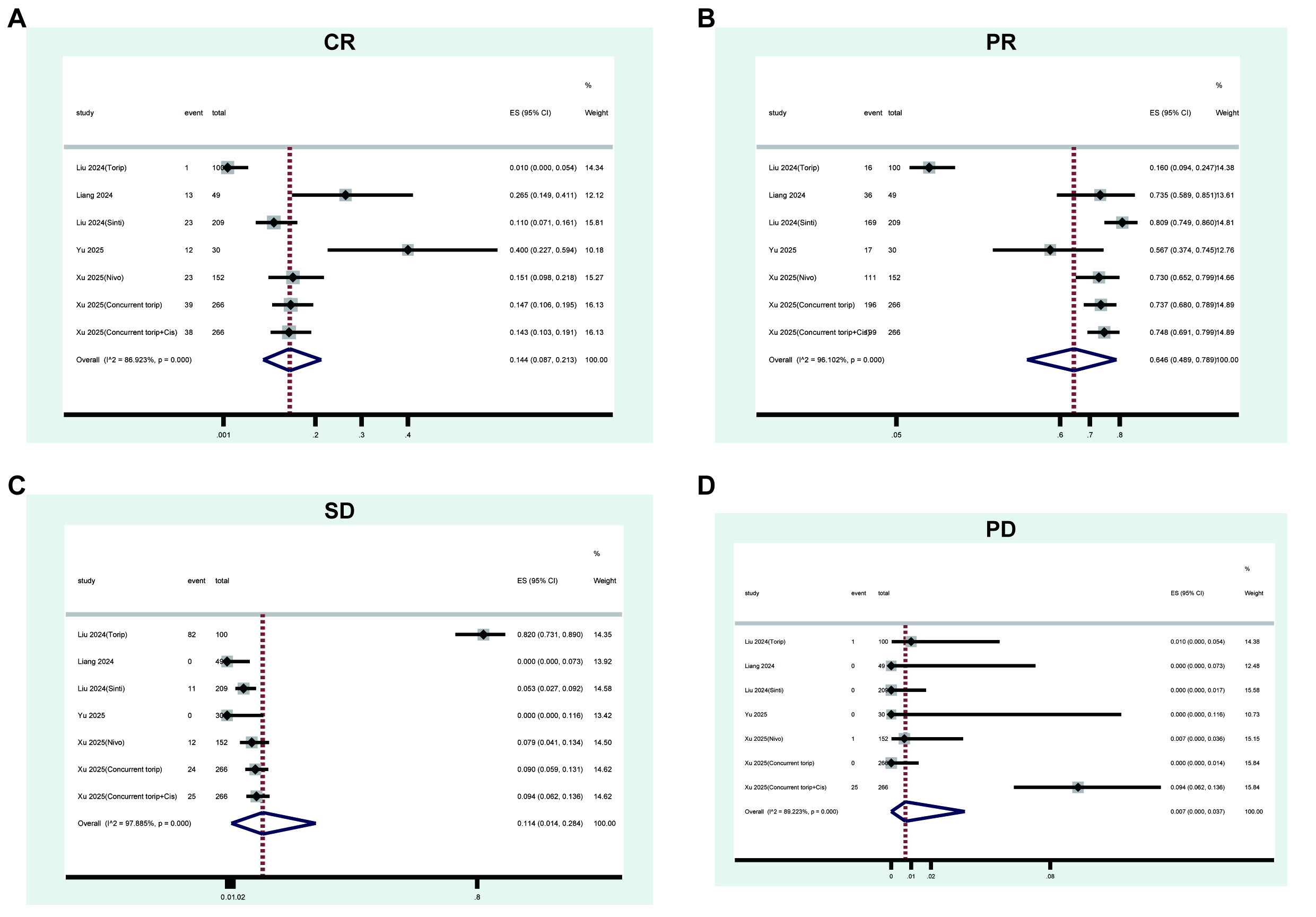

Supplement: Supplementary Figure 2 — Forest plots of tumor activity outcomes after neoadjuvant ICI-containing therapy. (A) CR, (B) PR, (C) SD, and (D) PD. LA-NPC, locoregionally advanced nasopharyngeal carcinoma; ICI, Immune checkpoint inhibitor; CR, complete response; PR, partial response; SD, stable disease; PD, progressive disease. [file Image2.tif]

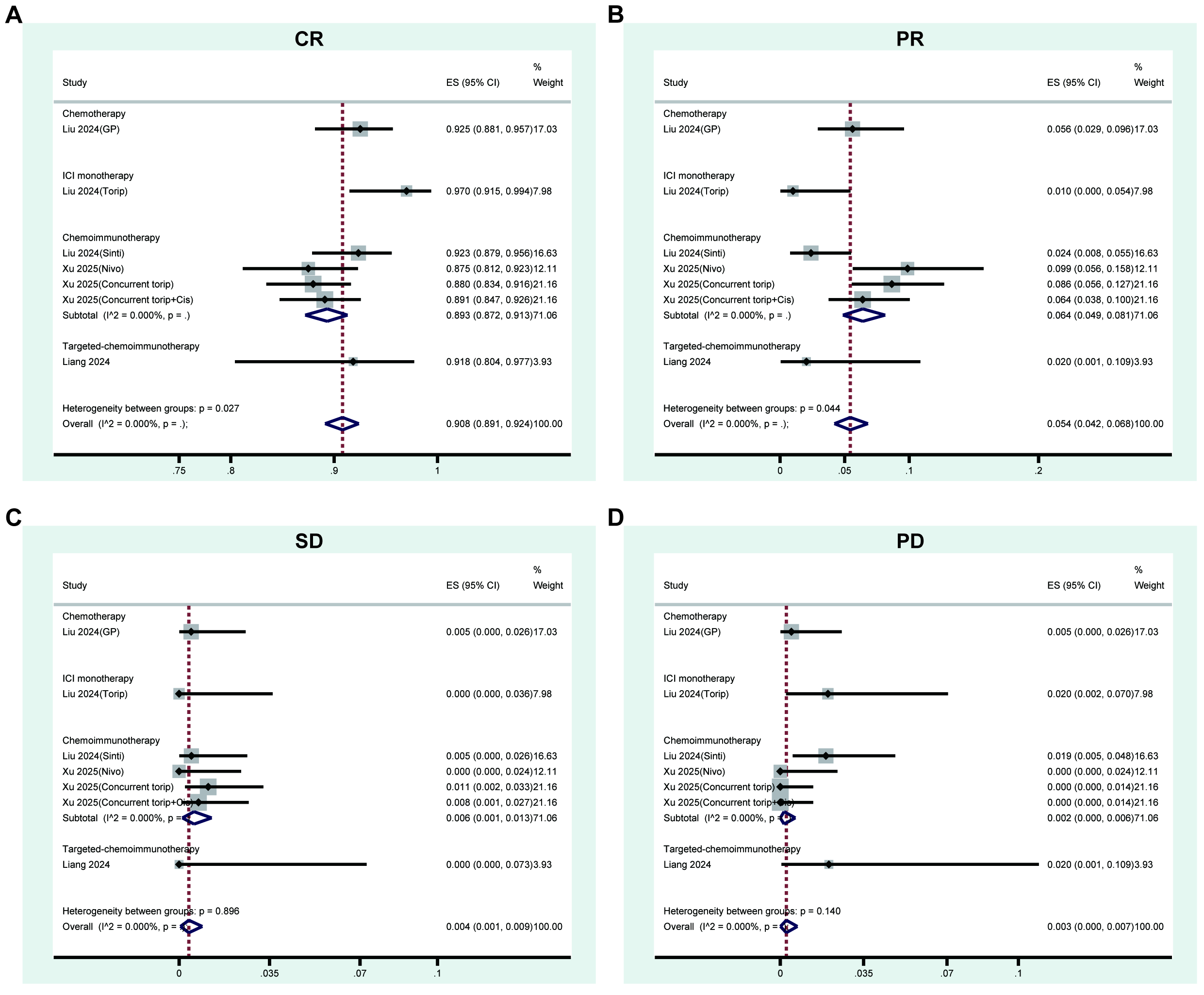

Supplement: Supplementary Figure 3 — Exploratory subgroup forest plots of tumor activity outcomes after completion of the full treatment regimen, stratified by neoadjuvant treatment regimen. (A) CR, (B) PR, (C) SD, and (D) PD. LA-NPC, locoregionally advanced nasopharyngeal carcinoma; CR, complete response; PR, partial response; SD, stable disease; PD, progressive disease. [file Image3.tif]

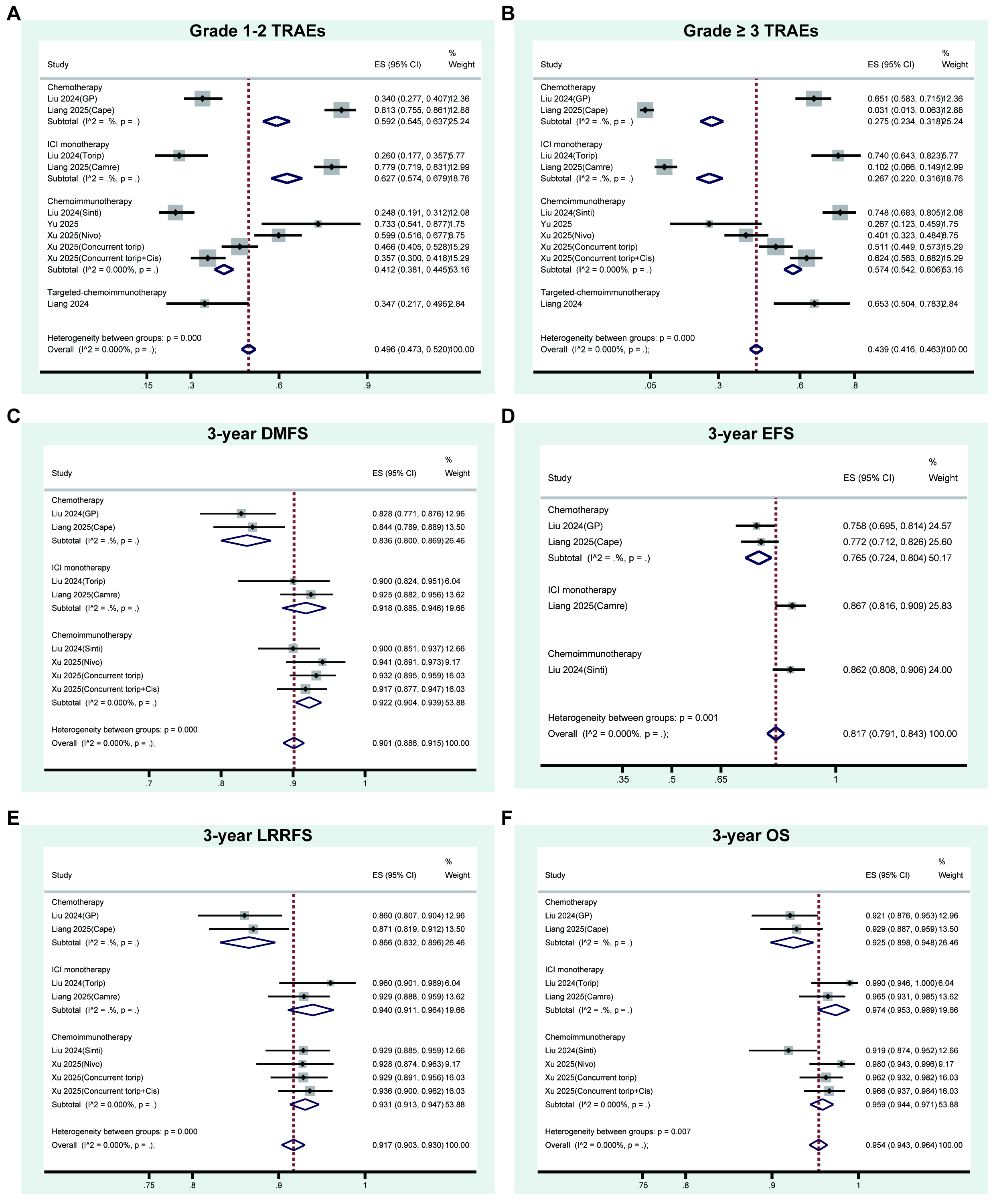

Supplement: Supplementary Figure 4 — Exploratory subgroup forest plots of safety and survival outcomes, stratified by neoadjuvant treatment regimen. (A) Grade 1–2 TRAEs, (B) grade ≥3 TRAEs, (C) 3-year DMFS, (D) 3-year EFS, (E) 3-year LRRFS, and (F) 3-year OS. LA-NPC, locoregionally advanced nasopharyngeal carcinoma; TRAEs, treatment-related adverse events; OS, overall survival; LRRFS, locoregional recurrence-free survival; DMFS, distant metastasis-free survival; EFS, event-free survival. [file Image4.tif]

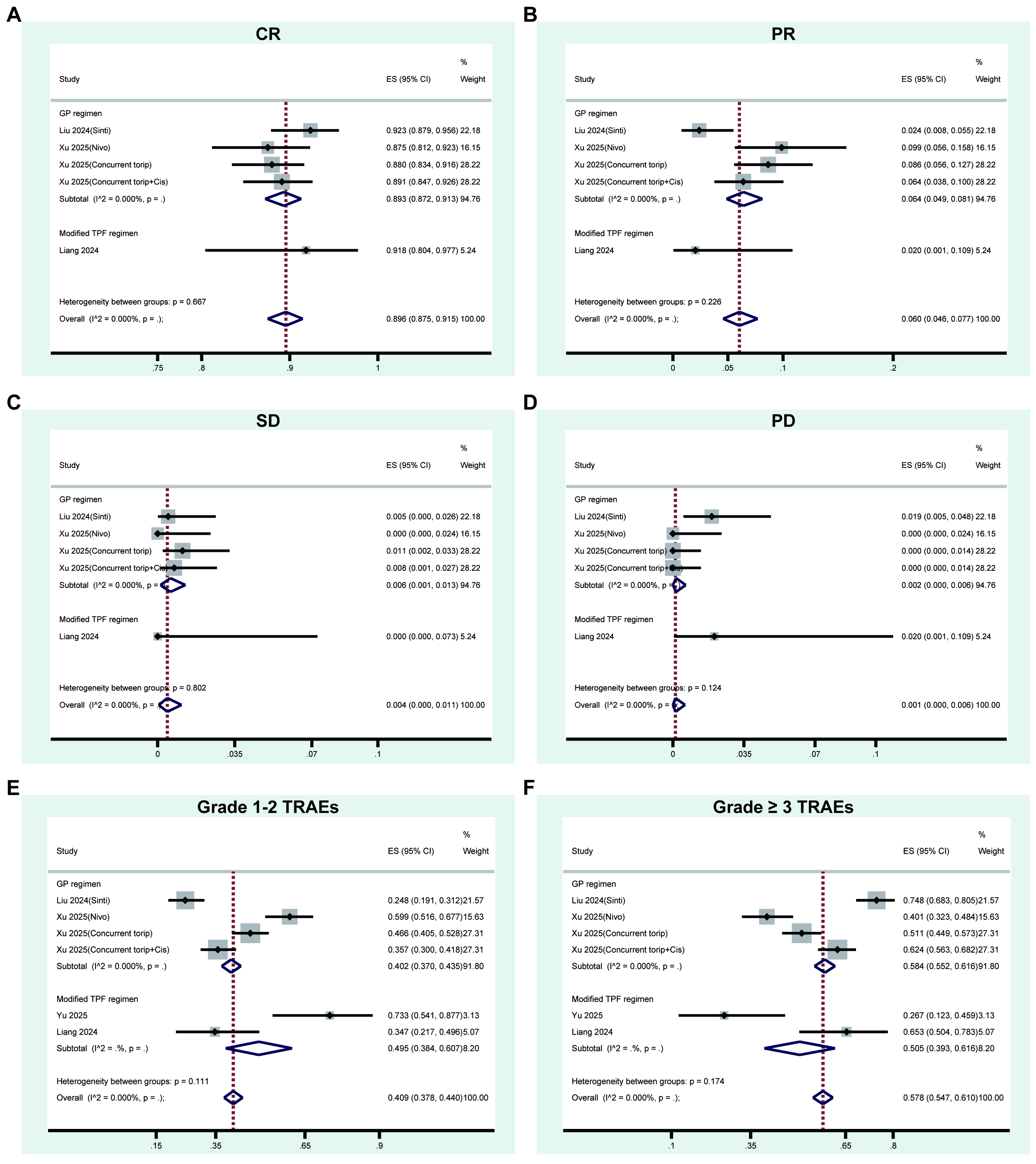

Supplement: Supplementary Figure 5 — Exploratory subgroup forest plots of tumor activity and safety outcomes after completion of the full treatment regimen, stratified by neoadjuvant chemotherapy backbone. (A) CR, (B) PR, (C) SD, (D) PD, (E) grade 1–2 TRAEs, and (F) grade ≥3 TRAEs. LA-NPC, locoregionally advanced nasopharyngeal carcinoma; CR, complete response; PR, partial response; SD, stable disease; PD, progressive disease. [file Image5.tif]

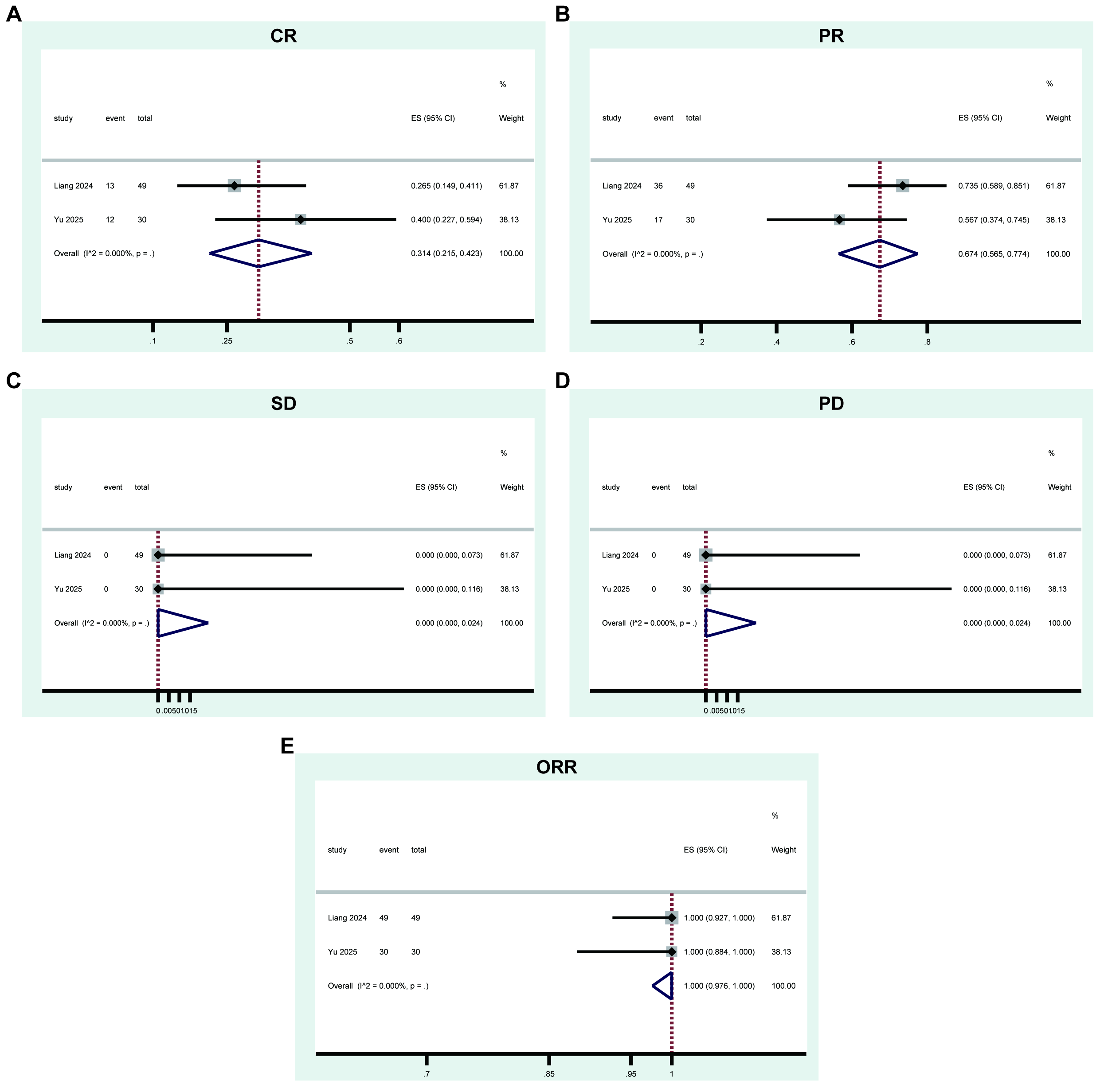

Supplement: Supplementary Figure 6 — Forest plots of tumor activity outcomes in patients with LA-NPC treated with camrelizumab-containing strategies. (A) CR, (B) PR, (C) SD, and (D) PD after neoadjuvant ICI-containing therapy; (E) ORR after completion of the full treatment regimen. LA-NPC, locoregionally advanced nasopharyngeal carcinoma; CR, complete response; PR, partial response; SD, stable disease; PD, progressive disease; ORR, objective response rate. [file Image6.tif]

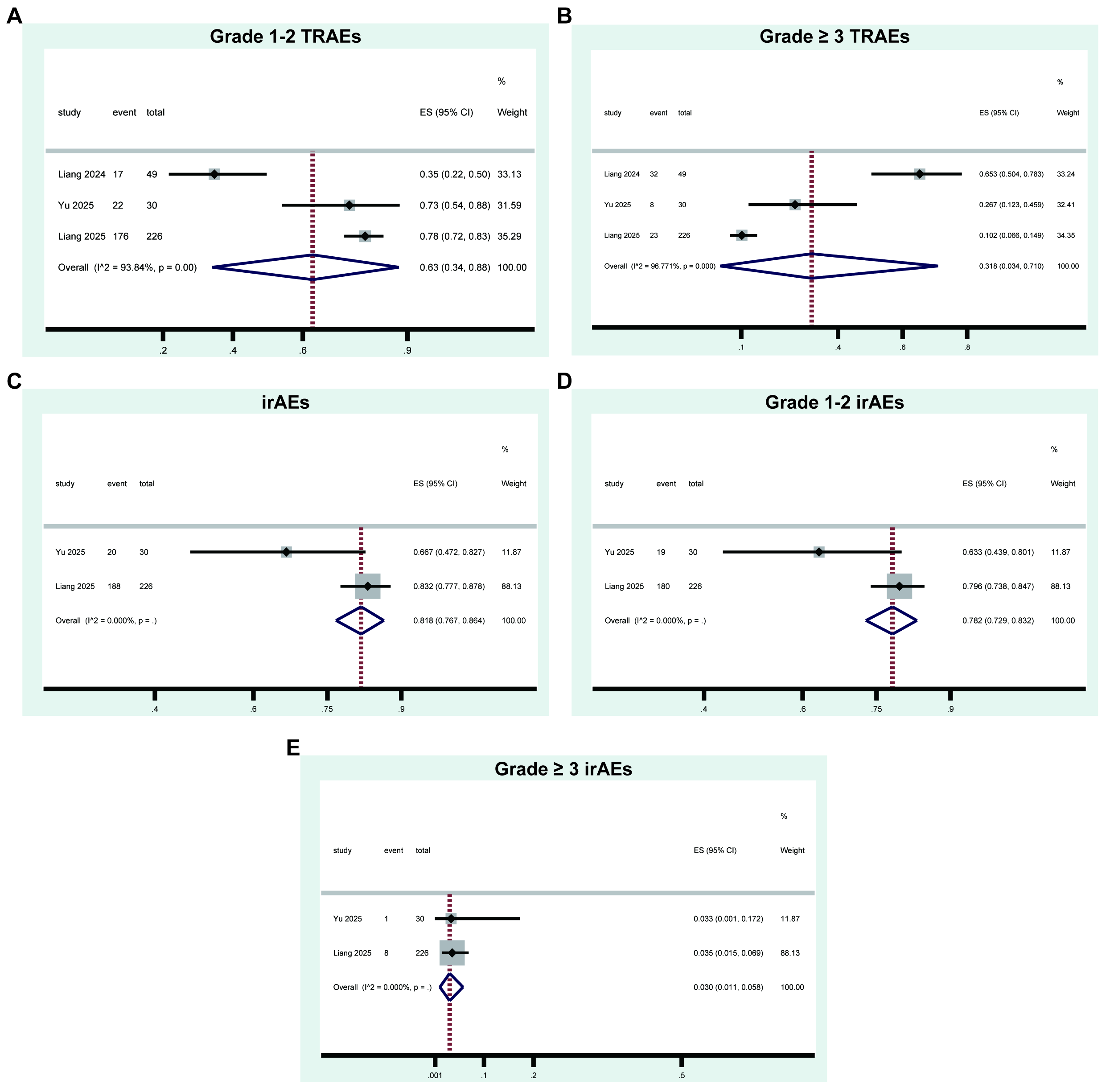

Supplement: Supplementary Figure 7 — Forest plots of regimen-level and immune-specific safety outcomes in patients with LA-NPC treated with camrelizumab-containing strategies. (A) Grade 1–2 TRAEs, (B) grade ≥3 TRAEs, (C) irAEs, (D) grade 1–2 irAEs, and (E) grade ≥3 irAEs. LA-NPC, locoregionally advanced nasopharyngeal carcinoma; TRAEs, treatment-related adverse events; irAEs, immune-related adverse events. [file Image7.tif]

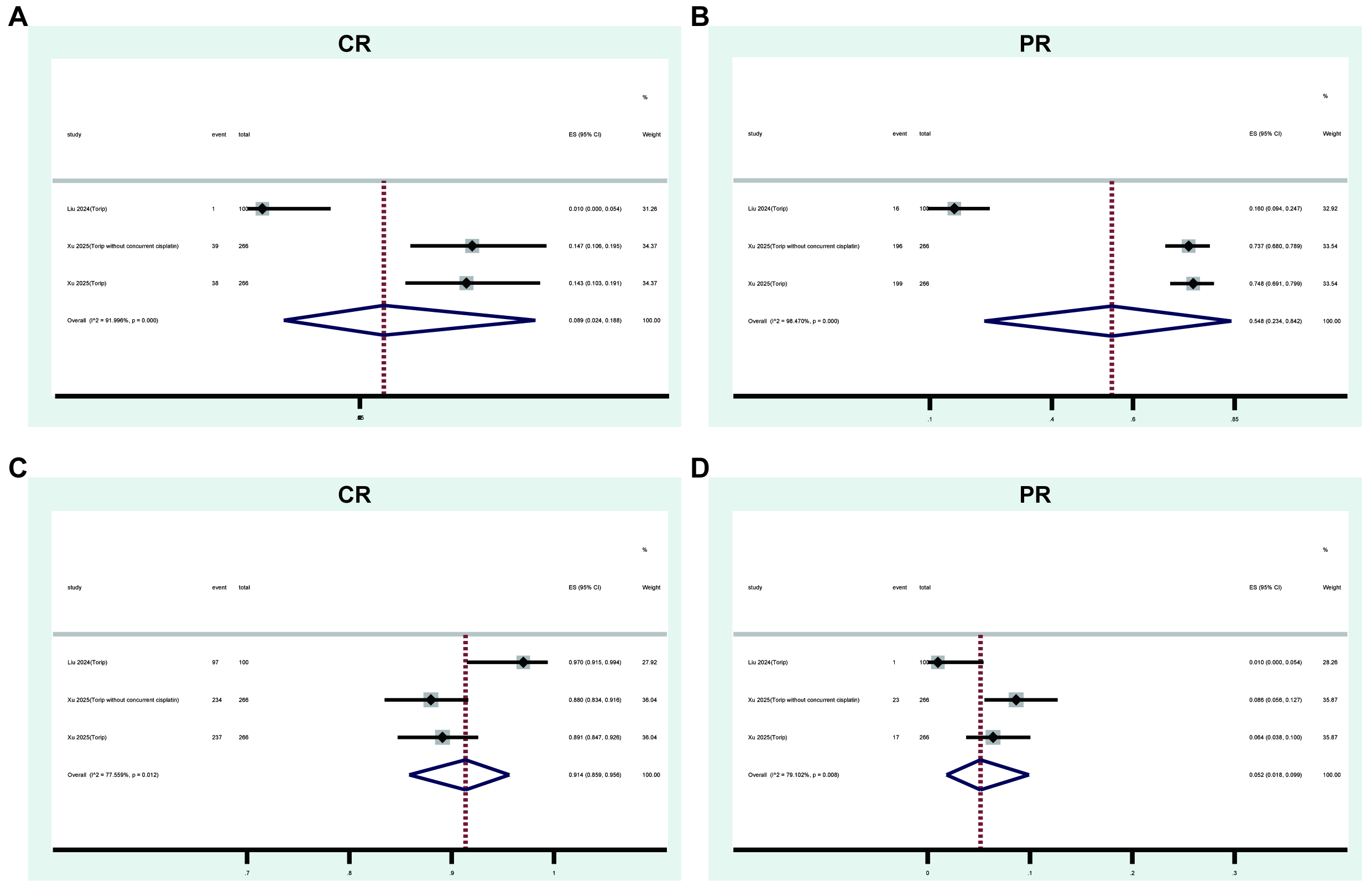

Supplement: Supplementary Figure 8 — Forest plots of tumor activity outcomes in patients with LA-NPC treated with toripalimab-containing strategies. (A) CR and (B) PR after neoadjuvant ICI-containing therapy; (C) CR and (D) PR after completion of the full treatment regimen. LA-NPC, locoregionally advanced nasopharyngeal carcinoma; CR, complete response; PR, partial response. [file Image8.tif]

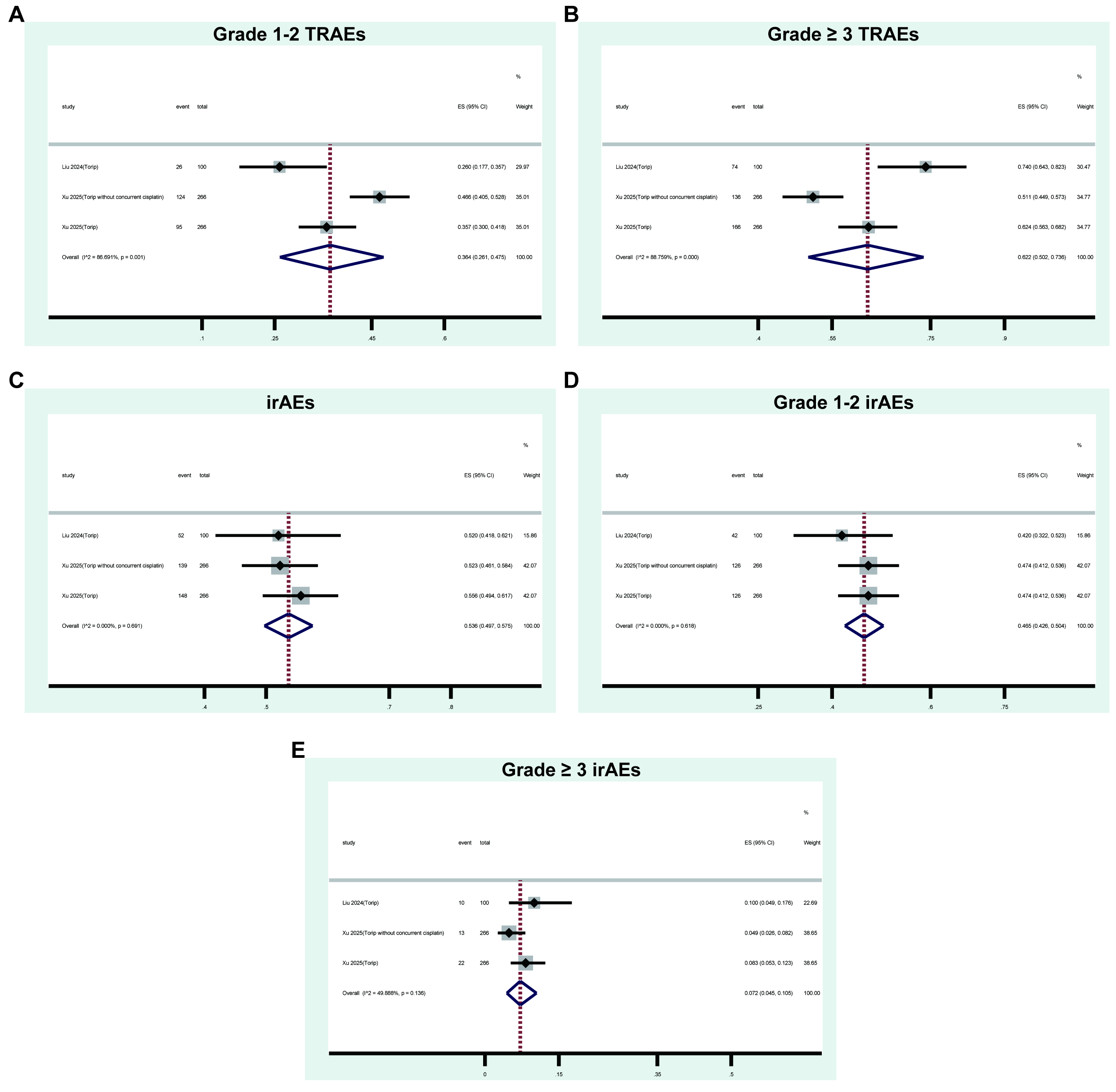

Supplement: Supplementary Figure 9 — Forest plots of regimen-level and immune-specific safety outcomes in patients with LA-NPC treated with toripalimab-containing strategies. (A) Grade 1–2 TRAEs, (B) grade ≥3 TRAEs, (C) irAEs, (D) grade 1–2 irAEs, and (E) grade ≥3 irAEs. LA-NPC, locoregionally advanced nasopharyngeal carcinoma; TRAEs, treatment-related adverse events; irAEs, immune-related adverse events. [file Image9.tif]

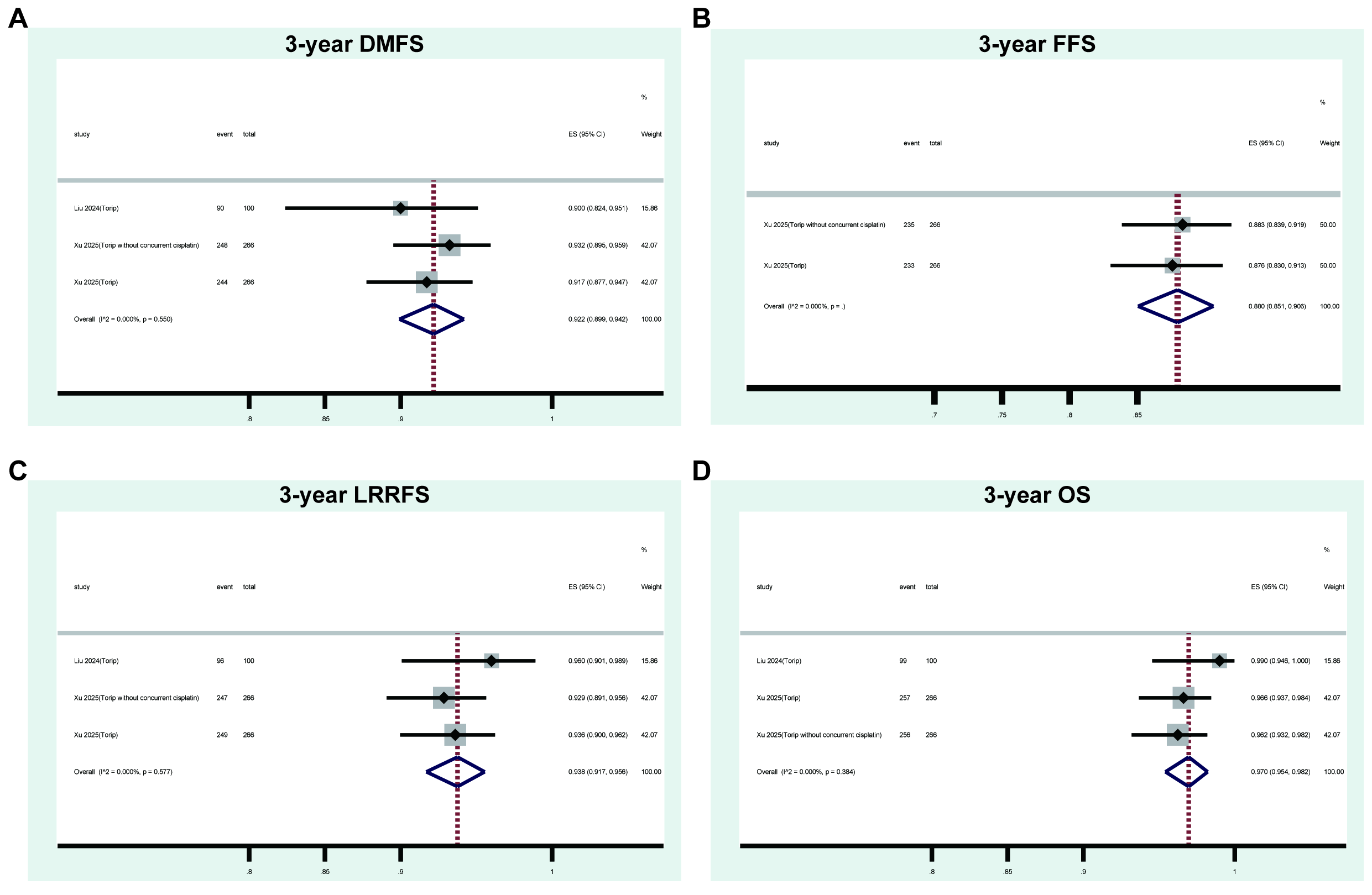

Supplement: Supplementary Figure 10 — Forest plots of survival outcomes in patients with LA-NPC treated with toripalimab-containing strategies. (A) 3-year DMFS, (B) 3-year FFS, (C) 3-year LRRFS, and (D) 3-year OS. LA-NPC, locoregionally advanced nasopharyngeal carcinoma; OS, overall survival; LRRFS, locoregional recurrence-free survival; DMFS, distant metastasis-free survival; FFS, failure-free survival. [file Image10.tif]

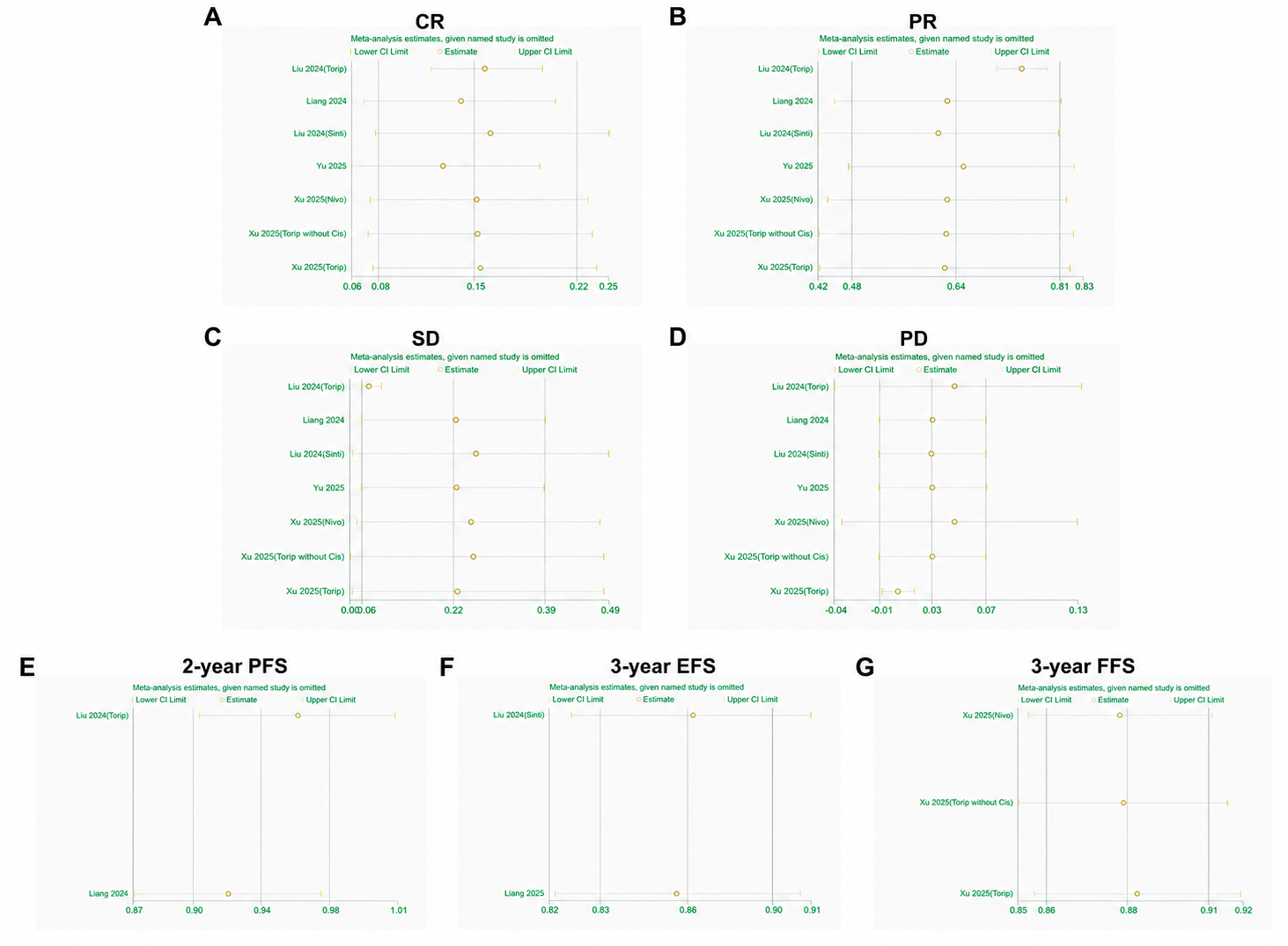

Supplement: Supplementary Figure 11 — Sensitivity analyses of pooled tumor activity and survival estimates in patients with LA-NPC treated with ICI-containing strategies. (A) CR, (B) PR, (C) SD, and (D) PD after neoadjuvant therapy; (E) 2-year PFS, (F) 3-year EFS, and (G) 3-year FFS. LA-NPC, locoregionally advanced nasopharyngeal carcinoma; CR, complete response; PR, partial response; SD, stable disease; PD, progressive disease; PFS, progression-free survival; EFS, event-free survival; FFS, failure-free survival. [file Image11.tif]

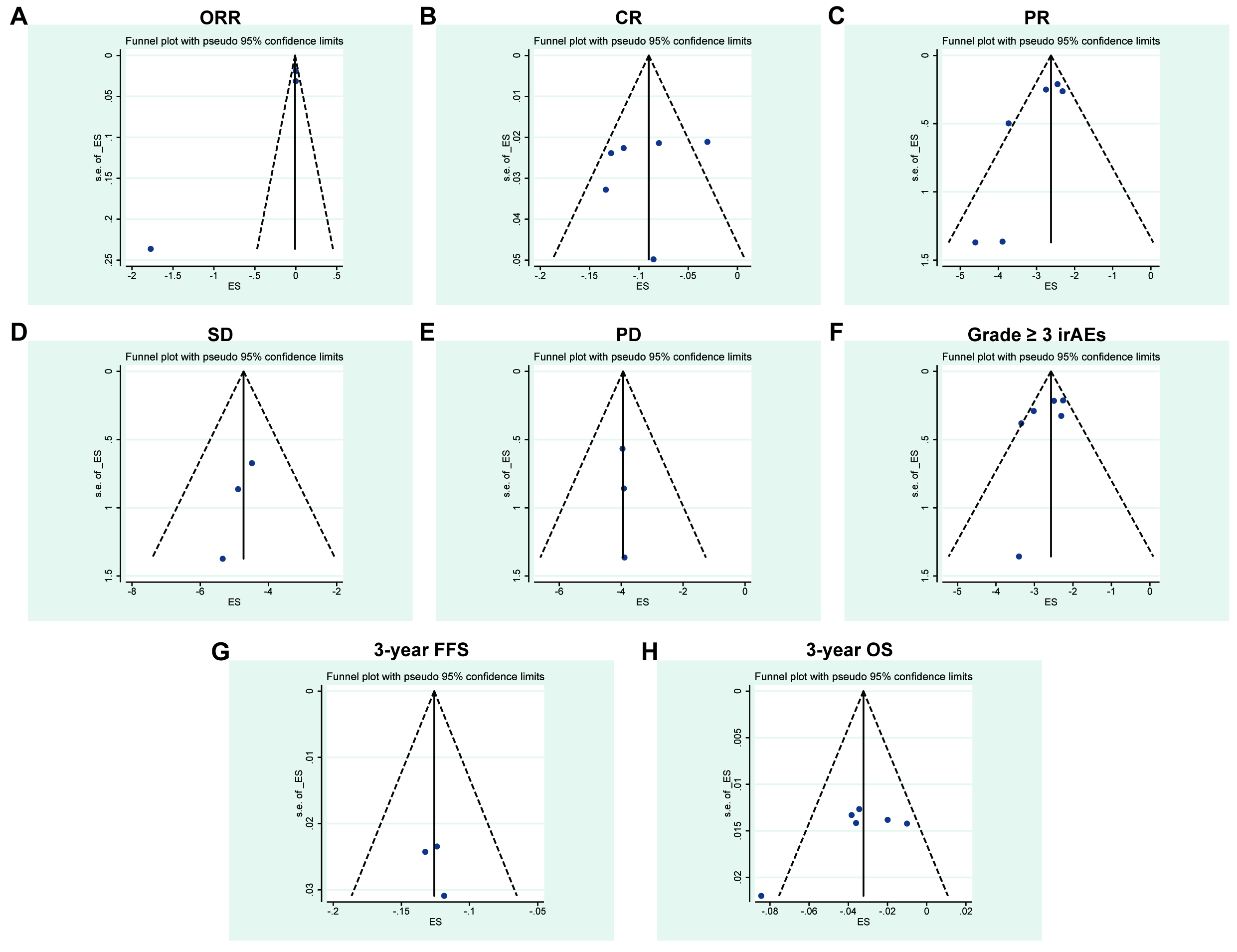

Supplement: Supplementary Figure 12 — Egger’s tests for small-study effects in pooled estimates. (A) ORR after neoadjuvant therapy, (B) CR, (C) PR, (D) SD, and (E) PD after completion of the full treatment regimen; (F) grade ≥3 irAEs, (G) 3-year FFS, and (H) 3-year OS. CR, complete response; PR, partial response; SD, stable disease; PD, progressive disease; irAEs, immune-related adverse events; OS, overall survival; FFS, failure-free survival. [file Image12.tif]
