# Supplementary material for: Immune checkpoint inhibitor integration in locoregionally advanced nasopharyngeal carcinoma: a prospective evidence synthesis on efficacy, safety, and therapeutic optimization
Source: Front Immunol. 2026 Jun 26;17:1840292. doi: 10.3389/fimmu.2026.1840292 (PMC13350252; doi:10.3389/fimmu.2026.1840292)
Supplement: Supplementary Table 1 — Detailed search strategy used in this systematic review and meta-analysis. [file DataSheet1.docx]

**Supplementary table 1: Search strategy in our meta-analysis.**

1. Search word associated with tumor: (Nasopharyngeal Neoplasms) OR (Nasopharyngeal Neoplasm) OR (Neoplasm, Nasopharyngeal) OR (Neoplasms, Nasopharyngeal) OR (Nasopharynx Neoplasms) OR (Nasopharynx Neoplasm) OR (Neoplasm, Nasopharynx) OR (Neoplasms, Nasopharynx) OR (Cancer of Nasopharynx) OR (Nasopharynx Cancers) OR (Nasopharyngeal Cancer) OR (Cancer, Nasopharyngeal) OR (Cancers, Nasopharyngeal) OR (Nasopharyngeal Cancers) OR (Nasopharynx Cancer) OR (Cancer, Nasopharynx) OR (Cancers, Nasopharynx) OR (Cancer of the Nasopharynx).
2. Search word associated with immunotherapy: (Immunotherapy) OR (Chemoimmunotherapy) OR (Chemo-immunotherapy) OR (Immune Checkpoint Inhibitors) OR (Checkpoint Inhibitors, Immune) OR (Immune Checkpoint Blockers) OR (Checkpoint Blockers, Immune) OR (Immune Checkpoint Inhibitor) OR (Checkpoint Inhibitor, Immune) OR (CTLA-4 Inhibitors) OR (CTLA 4 Inhibitors) OR (Cytotoxic T-Lymphocyte-Associated Protein 4 Inhibitors) OR (Cytotoxic T Lymphocyte Associated Protein 4 Inhibitors) OR (Cytotoxic T-Lymphocyte-Associated Protein 4 Inhibitor) OR (Cytotoxic T Lymphocyte Associated Protein 4 Inhibitor) OR (CTLA-4 Inhibitor) OR (CTLA 4 Inhibitor) OR (PD-1 Inhibitors) OR (PD 1 Inhibitors) OR (Programmed Cell Death Protein 1 Inhibitor) OR (Programmed Cell Death Protein 1 Inhibitors) OR (PD-1 Inhibitor) OR (Inhibitor, PD-1) OR (PD 1 Inhibitor) OR (Immune Checkpoint Blockade) OR (Checkpoint Blockade, Immune) OR (Immune Checkpoint Inhibition) OR (Checkpoint Inhibition, Immune) OR (PD-L1 Inhibitors) OR (PD L1 Inhibitors) OR (Programmed Death-Ligand 1 Inhibitors) OR (Programmed Death Ligand 1 Inhibitors) OR (PD-L1 Inhibitor) OR (PD L1 Inhibitor) OR (PD-1-PD-L1 Blockade) OR (Blockade, PD-1-PD-L1) OR (PD 1 PD L1 Blockade).
